# Hijacking the Host Clock: A Nematode Effector Antagonizes Soybean Circadian Defense and Translation Control

**DOI:** 10.1002/advs.202518591

**Published:** 2026-05-07

**Authors:** Xingwei Wang, Yufeng Xu, Yanfei Hu, Lijun Cao, Ru Jiang, Changtian Chen, Rick Masonbrink, Thomas Maier, Yuchen Tu, Yabo Shi, Enhui Liu, Lingan Kong, Chan Guo, Wei Zhao, Peng Shi, Wenzhen Du, Andrew Severin, Thomas Baum, Deliang Peng, Huan Peng, Mian Zhou, Wei Wang

**Affiliations:** ^1^ State Key Laboratory for Gene Function and Modulation Research School of Life Sciences Peking University Beijing China; ^2^ Center For Life Sciences Beijing China; ^3^ State Key Laboratory of Agricultural and Forestry Biosecurity College of Plant Protection Fujian Agriculture and Forestry University Fuzhou China; ^4^ College of Life Sciences Capital Normal University Beijing China; ^5^ Beijing Key Laboratory of Plant Gene Resources and Biotechnology for Carbon Reduction and Environmental Improvement Beijing China; ^6^ Department of Plant Pathology Entomology and Microbiology Iowa State University Ames Iowa USA; ^7^ College of Life Sciences and Oceanography Shenzhen University Shenzhen China; ^8^ State Key Laboratory for Biology of Plant Diseases and Insect Pests Institute of Plant Protection Chinese Academy of Agricultural Sciences Beijing China; ^9^ Genome Informatics Facility Office of Biotechnology Iowa State University Ames Iowa USA

**Keywords:** effector, plant immunity, soybean circadian clock, soybean cyst nematode, translation

## Abstract

The majority of commercially cultivated soybean cyst nematode (SCN)‐resistant soybean cultivars rely on the *Rhg1* locus to defend against SCN, the most economically destructive soybean root pathogen. The decline of Rhg1's effectiveness due to its continuous use creates an urgent need for alternative genetic sources to sustain SCN resistance. Although pathogen effectors are known to perturb host circadian clocks in other plant‐pathogen systems, whether and how plant‐parasitic nematodes interfere with the host clock to promote virulence remains unexplored. Here, we report that the soybean circadian clock gates rhythmic defense against SCN, and its core component, GmCCA1, plays a dual role in both circadian regulation and pathogen defense. Surprisingly, overexpression of *GmCCA1* disrupts circadian rhythms but confers sustained resistance against SCN by activating defense genes both within and beyond the *Rhg1* locus, including various *pathogenesis‐related* and *resistance* genes. On the pathogen side, we identify Hg4E02—an evolutionarily conserved effector in plant‐parasitic nematodes—as a transcriptional regulator that directly binds to the promoters of multiple clock genes via novel cis‐elements, suppressing their expression. Hg4E02 also represses defense gene expression to enhance virulence. Notably, we uncover a previously unknown layer of antagonism: GmCCA1 and Hg4E02 exert opposing effects on both defense genes and translation‐related genes. GmCCA1 inhibits translation and root growth, whereas Hg4E02 promotes translation, likely facilitating nutrient acquisition for the nematode. Collectively, our study reveals a novel mechanism by which a nematode effector directly targets the host circadian clock and translation machinery to promote parasitism, and proposes GmCCA1 as a promising engineering target for enhancing SCN resistance in soybean.

## Graphical abstract

Soybean employs its circadian clock, governed by GmCCA1, to rhythmically defend against soybean cyst nematodes. The pathogen retaliates by secreting the effector Hg4E02, which hijacks the clock to suppress defense and co‐opt the host's translation machinery for nutrient acquisition. This study unveils a novel arms race, identifying key targets for engineering durable resistance.

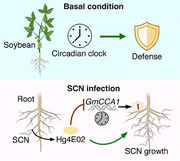

## 1 Introduction

The soybean cyst nematode (*Heterodera glycines*, SCN) is the most economically damaging pathogen of the soybean industry, causing over $1.5 billion loss annually in the USA alone [1, 2, 3]. The most economic and effective means to manage SCN is the use of SCN‐resistant soybean cultivars, which heavily rely on the *Resistance to Heterodera glycines 1* (*Rhg1*) locus. *Rhg1* accounts for the SCN resistance in over 95% of commercially cultivated SCN‐resistant northcentral American soybean cultivars [4]. However, the widespread and continuous use of the *Rhg1* locus is resulting in a decline in its effectiveness [5]. Given the lack of genetic diversity in currently deployed SCN‐resistant soybean cultivars and the waning effectiveness of known resistance loci, the discovery of alternative loci associated with enhanced SCN resistance is required to assure sustainable SCN resistance of commercial cultivars.

As an obligate root pathogen, SCN only feeds on live root cells. It can be envisioned that this intimate relationship may result in an interplay between soybean and SCN diurnal and/or circadian rhythms, governing soybean defense and SCN offense. The plant circadian clock could be a place of strategic importance contested by both the defender and the offender. Previous studies revealed that the plant circadian clock regulates rhythmic defense against bacterial, fungal, and oomycete pathogens, indicating that the plant displays varying immunity levels at different times of the day [6]. However, these studies primarily focused on the interplay between the model plant *Arabidopsis* and foliar pathogens [7]. How the circadian clock of crop plants interfaces with pathogens of economic importance remains largely unknown. Even less is known about whether and how the root circadian clock of crop plants interacts with root pathogens. However, the rhizosphere is where an extraordinary diversity of pathogens resides, and roots are at the forefront of the battle against soil‐borne pathogens.

In this study, we found that soybean displays both diurnal and circadian rhythms in resistance against SCN, which are dominated by the soybean circadian clock. However, as a successful obligate pathogen, SCN has gained the upper hand as it deploys Hg4E02, an effector with transcriptional regulatory activity to directly bind to several soybean clock gene promoters and suppress expression of genes including *Glycine max CIRCADIAN CLOCK ASSOCIATED 1* (*GmCCA1*, GLYMA_03G261800), *J*/*Glycine max EARLY FLOWERING 3a* (*GmELF3a*, GLYMA_04G050200) and *Glycine max PSEUDO‐RESPONSE REGULATOR 5*a (*GmPRR5a*, GLYMA_04G228300). Hg4E02 also suppresses defense gene expression to enhance SCN virulence. We performed RNA‐seq experiments and SCN infection assays to validate the role of GmCCA1 as a novel SCN‐resistance gene, which activates the well‐known resistance‐related genes in the *Rhg1* locus as well as various pathogenesis‐related (*PR*) genes and resistance (*R*) genes. Interestingly, GmCCA1 and Hg4E02 have opposing effects on transcriptional regulation of the soybean defense genes and translation‐related genes. Hg4E02‐mediated manipulation of plant circadian clocks may be a conserved invasion strategy, as its homologs could be identified in diverse plant‐parasitic nematodes. The molecular interaction between the soybean root circadian clock and SCN may lead to the discovery of uncharted routes for breeding new SCN‐resistant soybean cultivars.

## 2 Results

### 2.1 Soybean Circadian Clock Gates Defense Against SCN

Plants display different defense levels at different times of the day against multiple kinds of pathogens, including bacterial, fungal, and oomycete pathogens, which are regulated by the plant circadian clock [6]. This phenomenon is known as circadian clock gated defense. However, nearly all the previous studies on gated defense use the model plant *Arabidopsis*. Whether crops have a similar defense regulatory mechanism remains unknown. As an obligate biotrophic pest, SCN has an intimate interaction with its host, soybean. Therefore, we wondered whether the soybean circadian clock gates defense responses against SCN.

**FIGURE 1:**
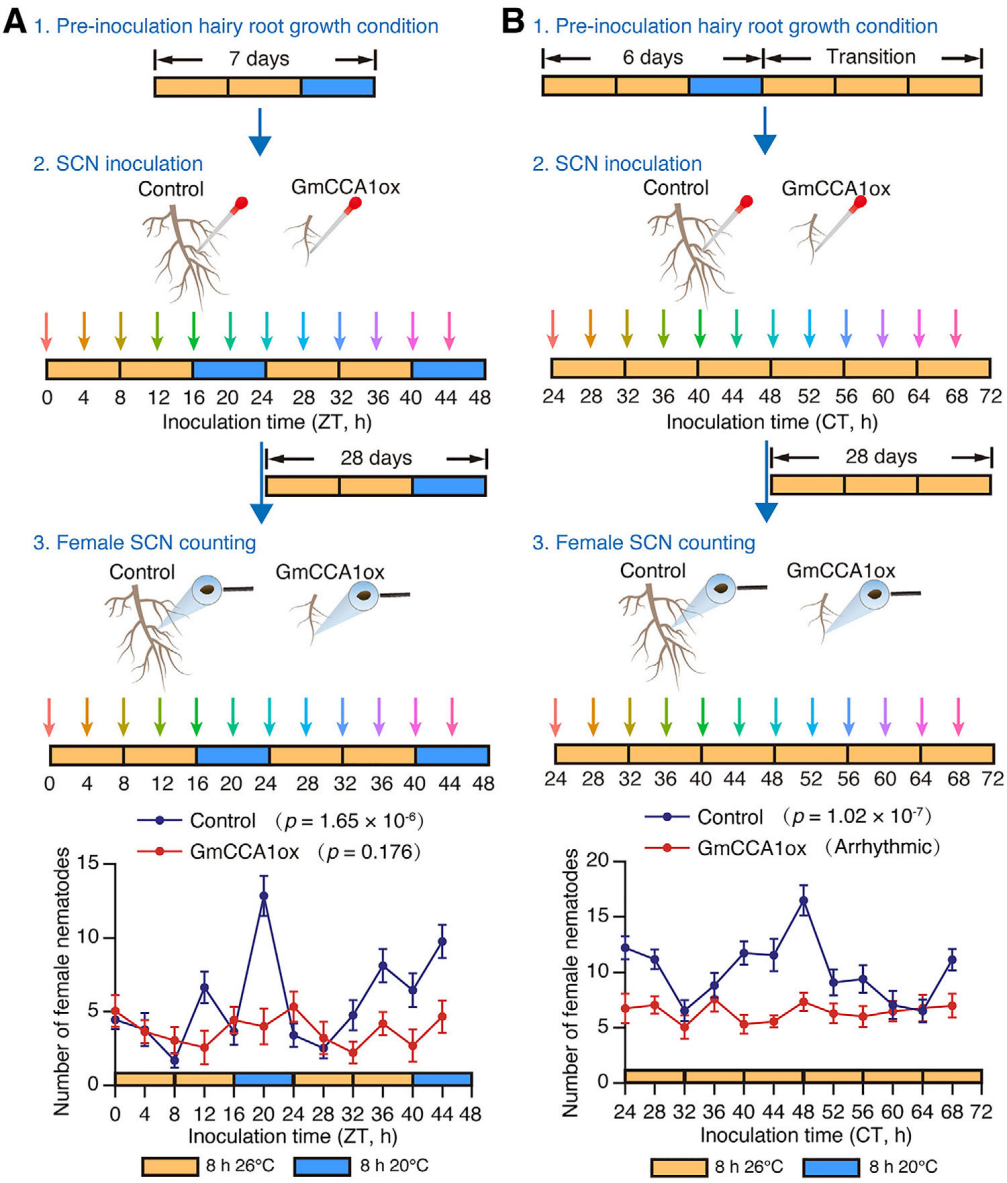
Soybean circadian clock gates defense against SCN. A‐B) Illustration (top) and quantification (bottom) of the time‐course SCN infection assays of Control and *GmCCA1* overexpression (GmCCA1ox) soybean hairy roots under the diurnal (A) or circadian (B) conditions. The data are shown as mean ± SEM (n = 7 ‐ 16 biological replicates). The oscillation robustness was derived through nonlinear regression analyses and *F*‐tests using linear trends as null hypotheses. Control showed significant resistance rhythms under both diurnal (*p* = 1.65 × 10^−6^) and circadian (*p* = 1.02 × 10^−7^) conditions. GmCCA1ox did not show robust resistance rhythms under either the diurnal (*p* = 0.176) or circadian conditions (unable to fit a cosine wave, the *p* value is not available). ZT, zeitgeber time. CT, circadian time.

Since SCN specifically infects soybean roots, soybean hairy roots have been widely used as an efficient and effective system to generate genetic modification to study SCN‐soybean interaction [8]. As a precursor to our studies that used roots taken from intact plants, we first used soybean hairy root system to perform a 4 h‐interval time‐course SCN infection assays under both diurnal and circadian conditions (Figure 1). For the diurnal condition, control soybean hairy roots (wild‐type soybean hairy root expressing empty vector) were first grown under the diurnal condition (16 h 26°C / 8 h 20°C) for 7 days. Starting from the dawn of the eighth day, SCN inoculation was performed every 4 h using different sets of hairy roots for 2 consecutive days. The inoculated hairy roots were kept under the diurnal condition for another 28 days, during which SCN completed their life cycle. Starting from the dawn of the 29^th^ day after inoculation, the number of female SCN was counted every 4 h for 2 consecutive days from the soybean hairy roots inoculated at the matching time of the day (Figure 1A). Nonlinear regression analysis and *F*‐test of the diurnal time‐course SCN infection data suggested a robust diurnal resistance rhythm (*p* = 1.65 × 10^−6^). Under the circadian condition (Figure 1B), control soybean hairy roots showed an even stronger circadian resistance rhythm (*p* = 1.02 × 10^−7^). These diurnal and circadian resistance rhythms are quite striking since the time interval between the adjacent SCN inoculations was 4 h, while the time interval between the inoculation and the final recording of the resistance phenotype was 28 days (672 h).

Theoretically, the resistance rhythms may reflect the defense rhythm of the soybean or the offense rhythm of SCN. However, if we consider the infection procedures of SCN, the observed diurnal and circadian resistance rhythms (Figure 1) are less likely to be solely determined by SCN. After inoculation of the soybean roots with the infective SCN juveniles, the nematodes can actually “hang around” for about one day before they start invasion. So, if there is a certain time of the day during which SCN juveniles are more likely to start infection, no matter when we inoculate the soybean with SCN juveniles during the day, they would “hang around” waiting for the preferred time of the day to start infection. Conceivably, this would abolish the rhythm we have observed. Furthermore, SCN has not been reported to show physiological rhythms. Since the number of infected female SCN was commonly used to represent the disease susceptibility of soybean, we performed a time‐course nematode hatching experiment to test the effects of the potential SCN circadian clock on SCN hatched number (Figure S1A). No significant differences were observed among the different hatching times, suggesting that the SCN circadian clock might not be the major determinant of the diurnal and circadian resistance differences of soybean. Therefore, we hypothesized that the diurnal and circadian resistance rhythms are likely to be governed by the soybean circadian clock.

To test whether these resistance rhythms are dependent on the soybean circadian clock, we generated soybean hairy roots overexpressing a soybean central circadian clock gene, *GmCCA1*. In *Arabidopsis*, overexpression of *AtCCA1* disrupts the circadian clock [9]. Through RT‐qPCR, we found that in the hairy roots overexpressing *GmCCA1*, the expression levels of *GmCCA1* were strongly induced and its oscillatory expression patterns were abolished under both diurnal and circadian conditions (Figure S1B, C). After confirming the destruction of the soybean circadian clock in the *GmCCA1* overexpression hairy roots, we performed time‐course SCN infection assays using *GmCCA1* overexpression hairy roots under the diurnal and circadian conditions (Figure 1). Both diurnal and circadian resistance rhythms were abolished in the *GmCCA1* overexpression hairy roots according to the nonlinear regression analyses and *F*‐tests. Therefore, the soybean rhythms in resistance against SCN that we observed are governed by the soybean circadian clock.

### 2.2 Circadian Clock Component GmCCA1 Promotes Defense Gene Expression and Suppresses Translation‐related Gene Expression

**FIGURE 2:**
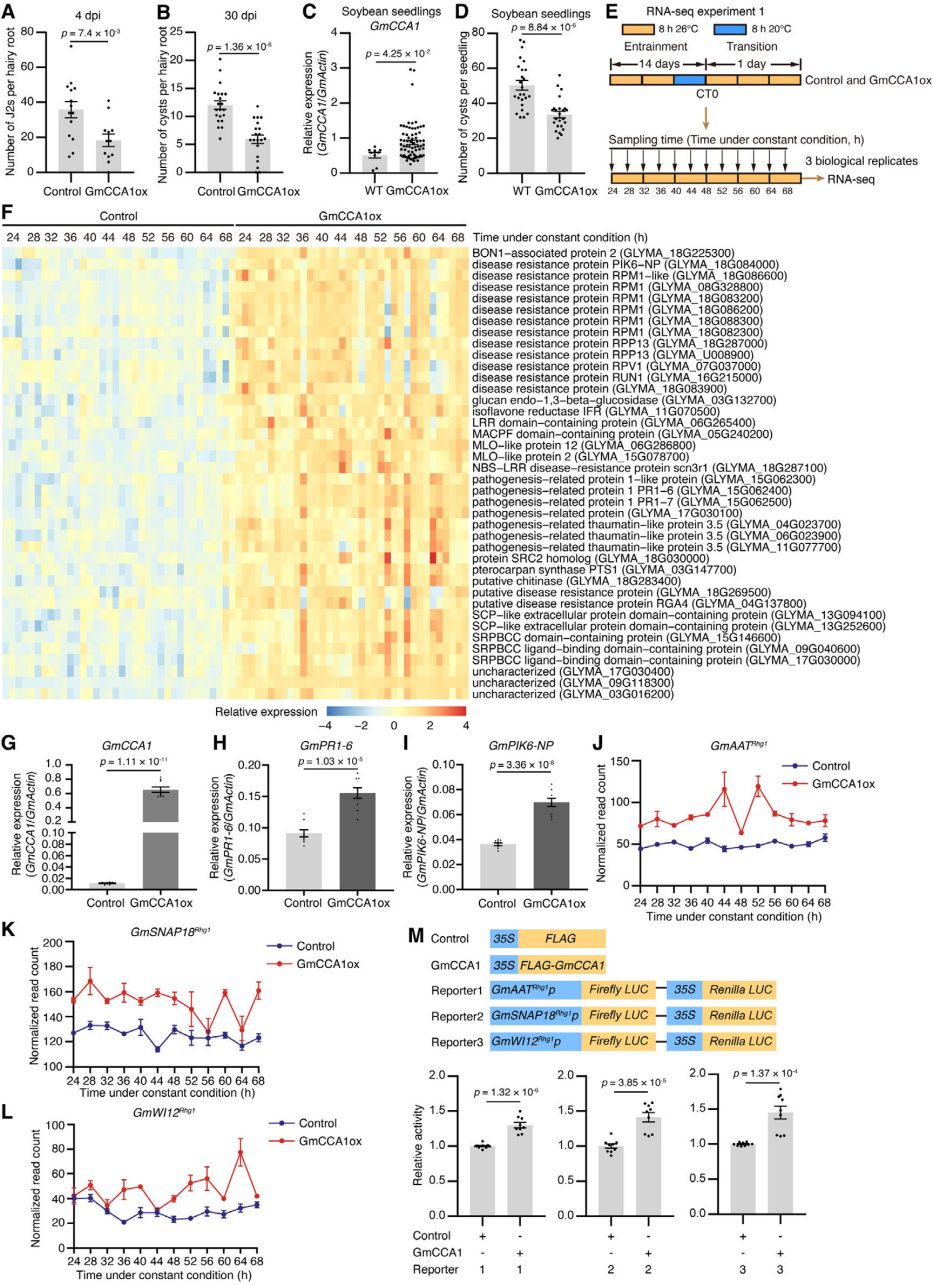
GmCCA1 promotes defense gene expression. (A) The number of penetrated J2s 4 days post inoculation (dpi) on each Control and *GmCCA1* overexpression (GmCCA1ox) soybean hairy root. The data are shown as mean ± SEM (*n* = 12–21 biological replicates). The *p* value was calculated using the Mann‐Whitney test. B) The number of cysts at 30 dpi on each Control and GmCCA1ox soybean hairy root. The data are shown as mean ± SEM (n = 12–21 biological replicates). The *p* value was calculated using the Mann‐Whitney test. (C) Quantitative real‐time PCR analysis of *GmCCA1* transcript abundance in WT and GmCCA1ox soybean seedlings at ZT2. The data are shown as mean ± SEM (*n* = 3 biological samples for Control, 24 biological samples for GmCCA1ox, each biological sample has 3 technical replicates). The *p* value was calculated using two‐sided Student's *t*‐test. (D) The number of cysts on the roots of each Control and GmCCA1ox transgenic soybean seedling at 30 days after inoculation. The data are shown as mean ± SEM (*n* = 25 for Control, 24 for GmCCA1ox). The *p* value was calculated using the Mann‐Whitney test. (E) Illustration of the experimental design of the circadian time‐course RNA‐seq of Control and GmCCA1ox soybean hairy roots. Arrows indicate 12 sampling time points. Each time point has 3 biological replicates. CT, circadian time. (F) Expression heatmap of GmCCA1‐induced defense‐related genes in Control and GmCCA1ox samples. Data from RNA‐seq analysis. (G–I) Relative expression of *GmCCA1* (G), *GmPR1‐6* (H), and *GmPIK6‐NP* (I) in Control and GmCCA1ox soybean hairy roots verified by RT‐qPCR. The data are shown as mean ± SEM (*n* = 3 biological replicates with 3 technical replicates per sample). The *p* values were calculated using two‐sided Student's *t*‐test. (J–L) Normalized read counts of *GmAAT^Rhg1^* (J), *GmSNAP18^Rhg1^* (K), and *GmWI12^Rhg1^* (L) in Control and GmCCA1ox soybean hairy roots. The data are shown as mean ± SEM (*n* = 3 biological replicates). M) Dual‐luciferase assays performed using *Nicotiana benthamiana* leaves transiently co‐expressing FLAG‐GmCCA1 or FLAG (control) and the reporters driven by the promoter of *GmAAT^Rhg1^* (Reporter1), *GmSNAP18^Rhg1^* (Reporter2), or *GmWI12^Rhg1^* (Reporter3). The ratio of firefly luciferase and *Renilla* luciferase activities was calculated and normalized to the control. The data are shown as mean ± SEM (*n* = 3 biological replicates with 3 technical replicates per sample). The *p* values were calculated using two‐sided Student's *t*‐test.

Interestingly, while overexpression of *GmCCA1* in soybean hairy roots abolished the resistance rhythms, it significantly enhanced resistance against SCN regardless of the timing of inoculation, indicating that aside from its primary function in the circadian clock system, GmCCA1 may also positively regulate the defense against SCN (Figure 1). This enhanced SCN resistance of *GmCCA1* overexpression could already be observed as early as 4 days after SCN inoculation, similar to that observed at 30 days after SCN inoculation (Figure 2A,B).

To further support the conclusion that GmCCA1 positively regulates defense against SCN, we generated *GmCCA1* overexpression soybean seedlings. The expression of *GmCCA1* was elevated in these transgenic seedlings (Figure 2C), and these overexpression seedlings were significantly more resistant to SCN compared to WT seedlings (Figure 2D).

To explore the effects of *GmCCA1* overexpression on global transcriptome, we performed circadian time‐course RNA‐seq analysis (Figure 2E). Wild‐type soybean hairy roots overexpressing empty vector (Control) or *GmCCA1* (GmCCA1ox) were first entrained under a 16 h 26°C /8 h 20°C thermal cycle for 14 days, followed by a one‐day acclimation under the constant condition of 26°C. The samples were harvested every 4 h for 2 consecutive days. Three biological replicates used in this RNA‐seq experiment displayed sound reproducibility (Figure S2). Consistent with the RT‐qPCR data, RNA‐seq analysis also revealed the significantly elevated expression levels and abolished rhythmic expression pattern of *GmCCA1* in the *GmCCA1* overexpression hairy roots (Figure S3A). In addition to *GmCCA1*, the circadian rhythmic expression pattern of other central circadian clock genes was also significantly compromised (Figure S3B–L). We further performed molecular timetable analysis, a single‐time‐point analysis algorithm for analysis of global rhythm [10] using the RNA‐seq data (Figure S4). The molecular timetable analysis allows derivation of the global rhythm of each sample using a set of time‐indicating genes (Figure S4A). Nonlinear regression analyses fitted these curves to cosine waves for the estimation of the period, phase, and amplitude. *F*‐tests comparing the fitting to cosine waves and straight lines gave rise to the oscillation *p* values (an indicator of robustness of the circadian rhythm). In the radial plot showing the phase and ‐log_10_(*p*), the sample points located farther from the center of the circle have more robust circadian rhythms (Figure S4B). These analyses confirmed that *GmCCA1* overexpression drastically reduced the oscillation robustness for all the sampling time points and destroyed the soybean circadian clock completely.

Since GmCCA1ox is arrhythmic, we searched for the differentially expressed genes caused by overexpression of *GmCCA1* by disregarding the time factor. Through grouping all the samples according to their genotypes, followed by Wilcox tests, we identified 2,181 GmCCA1‐induced and 2,211 GmCCA1‐repressed genes. Gene ontology (GO) analysis of these GmCCA1‐induced genes found a significant enrichment of genes involved in defense responses and plant‐type hypersensitive response (HR) (Figure S5A; Supplementary Dataset 1). To better visualize the expression of these defense‐related genes, we plotted the expression heatmap of defense‐related genes that were upregulated in *GmCCA1* overexpression hairy roots compared to the control, which included *PR* genes like *PR1*‐homologous genes as well as *R* genes like *PIK6‐NP*, *Resistance to Pseudomonas syringae pv maculicola* 1 (*RPM1*), and *Recognition of Peronospora parasitica* 13 (*RPP13*) (Figure 2F). These R proteins have a nucleotide‐binding domain and leucine‐rich repeat (NLR) and play important roles in plant immunity [11]. To further confirm the expression of defense genes, we performed RT‐qPCR to analyze the expression of a representative *PR* gene, *GmPR1‐6*, and a representative *R* gene, *GmPIK6‐NP*, in the control and *GmCCA1* overexpression soybean hairy roots (Figures 2G‐I). High expression of these defense genes may contribute to the elevated resistance against SCN in *GmCCA1* overexpression.

Notably, the presence of 6 copies of soybean Membrane Attack Complex and Perforin (MACPF) domain‐containing proteins homologous to *Arabidopsis* Constitutive Active Defense 1 (CAD1), MACPF‐like 1 (MACP1), and MACPF‐like 2 (MACP2) in GmCCA1‐induced genes made the GO term, “plant‐type HR” significantly enriched (Figure S5B). In animals, upon establishment of the immune synapse, MACPF proteins trigger cell death by assembling into oligomeric pores on the cell surface, breaching the integrity of the targeted cells [12]. In plants, CAD1 and MACPF2 are also associated with programmed cell death [13, 14]. Since programmed cell death is a potent defense response against biotrophic pathogens, the upregulation of MACPF domain‐containing proteins might contribute to the SCN resistance of *GmCCA1* overexpression soybean hairy roots (Figure 2A).

Most commercially cultivated SCN‐resistant soybean cultivars carry the resistance alleles of the *Rhg1* locus [4]. *Rhg1*‐mediated SCN‐resistant soybean cultivars typically have 7‐10 copies of the *Rhg1* locus in their genomes, which correlates with the basal expression levels of the resistance‐related genes encoded in *Rhg1* and overall SCN resistance [15]. We wondered whether the enhanced SCN resistance of *GmCCA1* overexpression lines is also linked to the elevated expression of defense genes at the *Rhg1* locus. To test this, we examined the RNA‐seq data (Figure 2E) and discovered that GmCCA1 induced expression of putative *Amino Acid Transporter* (*GmAAT^Rhg1^*, GLYMA_18G022400), *Glycine max Soluble NSF Attachment Protein 18* (*GmSNAP18^Rhg1^*, GLYMA_18G022500), and *Glycine max Wound‐inducible Protein 12* (*GmWI12^Rhg1^*, GLYMA_18G022700), the three major resistance‐related genes encoded in the *Rhg1* locus (Figure 2J–L). Through dual‐luciferase assays using the promoters of *GmAAT^Rhg1^*, *GmSNAP18^Rhg1^*, and *GmWI12^Rhg1^*, we found that GmCCA1 could induce the expression of these genes (Figure 2M). Consistent with the regulatory effect of GmCCA1 on *GmAAT^Rhg1^*, *GmSNAP18^Rhg1^*, and *GmWI12^Rhg1^* in soybean hairy roots, the expression levels of these three genes exhibited a statistically significant linear relationship with the expression levels of *GmCCA1* in the *GmCCA1* overexpression soybean seedlings (Figure S5C). Therefore, the induction of the resistance‐related genes encoded in the *Rhg1* locus could be one of the contributors to GmCCA1‐mediated defense against SCN.

GO analysis of GmCCA1‐repressed genes identified significant enrichment of genes encoding large ribosomal subunits (Figure S5D; Supporting Information S2: Dataset 1), including *Glycine max Ribosomal Protein Large subunit 5* (*GmRPL5*, GLYMA_13G280300), *GmRPL10* (GLYMA_18G050600), *GmRPL32* (GLYMA_15G195500) and *GmRPL37* (GLYMA_13G357900) (Figure S5E–H), indicating that GmCCA1 may affect translation to regulate plant growth.

### 2.3 SCN Interferes With the Soybean Root Circadian Clock

As an obligate root pathogen, SCN only feeds on live root cells. While this intimate relationship leads to soybean circadian clock gated defense against SCN (Figure 1), SCN may also affect the soybean circadian clock to interfere with clock or clock gene‐mediated defense.

**FIGURE 3:**
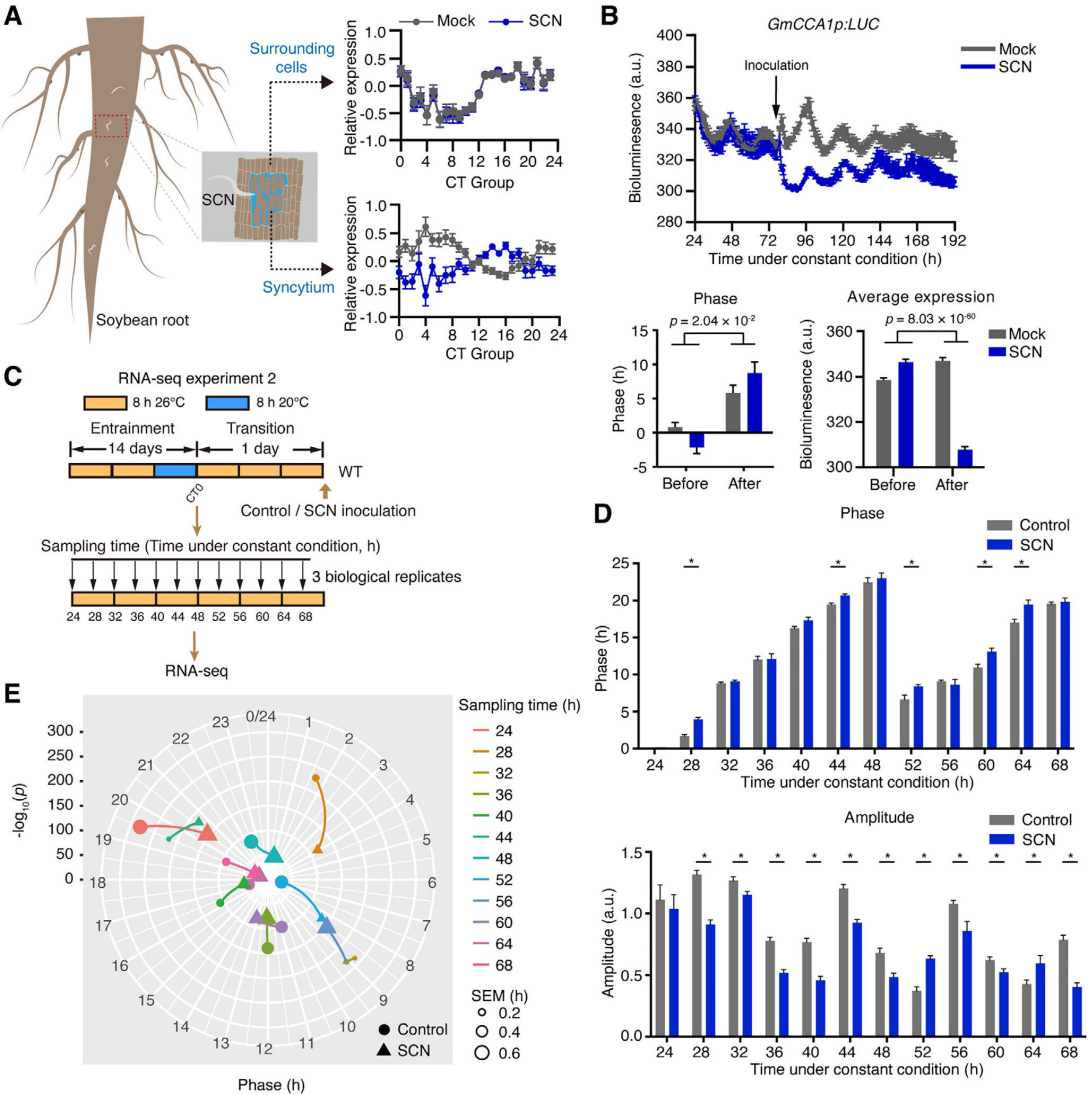
SCN interferes with the soybean root circadian clock. (A) Schematic diagram to compare global rhythm changes caused by SCN in the syncytium and surrounding cells. Normalized expression levels of time‐indicating genes are plotted upon Mock or SCN treatment. The data are shown as mean ± SEM (*n* = 3 biological replicates). CT, circadian time. The original datasets were obtained from previous studies [17, 18]. (B) Bioluminescence of transgenic hairy roots expressing *GmCCA1p:LUC*. Mock or SCN was inoculated at 76 h under the constant condition indicated by the arrow. The data are shown as mean ± SEM (*n* = 3 biological replicates). The bar graph shows the phase and average expression before and after inoculation. The *p* values were calculated using two‐way ANOVA. h, hour. a.u., arbitrary unit. (C) Illustration of the experimental design of the circadian time‐course RNA‐seq experiment 2. Arrows indicate 12 sampling time points. Each time point has 3 biological replicates. (D) Phase and amplitude changes caused by SCN at 12 sampling time points. The data are shown as mean + SEM (*n* = 3 biological replicates). The phase and amplitude of each sampling time point were calculated through the molecular timetable method. The asterisks represent statistically significant differences (*p* < 0.05, Holm‐Šídák's multiple comparisons test). (E) Radial plot showing the phase shift and robustness changes caused by SCN at each sampling time point. Phase is plotted as the angular coordinate. Robustness plotted as the radial distance is indicated by ‐log_10_(*p*), with a larger ‐log_10_(*p*) representing better oscillation. SEMs are indicated by the size of the circles (Control) or triangles (SCN).

SCN obtains nutrients from soybean roots through an enlarged, multi‐nucleated, and metabolically hyperactive feeding site called the syncytium [16]. Genome‐wide expression profiling of isolated syncytia and surrounding cells two days after SCN infection was reported [17, 18]. While these single‐time‐point expression profiles certainly do not have a sufficient time resolution to reveal the oscillatory expression pattern of circadian clock genes, they can be used to derive the global rhythm through the molecular timetable method [10]. Using the molecular timetable method that we previously established in soybean [10], we found that SCN significantly perturbed the global rhythm in syncytium but not in the surrounding cells (Figure 3A, Figure S6A,B). Similarly, sugar beet cyst nematode (BCN, *Heterodera schachtii*) also significantly perturbed the global rhythm in syncytial cells in *Arabidopsis* (Figure S6C). It should be emphasized that these results are just computational predictions, implying that SCN may perturb soybean circadian rhythm. Although syncytium‐specific changes were observed, SCN may affect the soybean circadian clock before the formation of syncytia. Furthermore, one soybean root can be simultaneously attacked by many infective SCN juveniles, resulting in the establishment of multiple feeding sites on a single root. Therefore, SCN's effect on the soybean circadian clock may also be documented at the tissue level.

To further test the influence of SCN on the temporal expression pattern of the soybean circadian clock genes, we generated transgenic soybean hairy roots harboring the luciferase coding sequence driven by the promoter of *GmCCA1*. Bioluminescence imaging of *GmCCA1p:LUC* suggested that SCN significantly repressed the average expression levels of *GmCCA1* (Figure 3B). This result demonstrates that the effect of SCN on soybean circadian clock is rapid, well before the formation of syncytia, and could indeed be recorded at the tissue level.

To enable a comprehensive view of SCN's effect on the soybean root circadian rhythm, we performed a circadian time‐course RNA‐seq analysis of soybean hairy roots with control or SCN inoculation under circadian conditions (Figure 3C). This RNA‐seq experiment was conducted at the same time as the RNA‐seq experiment 1 (Figure 2E) under the same condition and shared the control samples. Control or SCN inoculation was performed at the subjective morning, and the samples were harvested every 4 h for 2 consecutive days. Three biological replicates were obtained for RNA‐seq analysis. The heatmaps of the pairwise Pearson correlation of these 72 samples suggested good reproducibility between biological replicates (Figure S6D). Through nonlinear regression and statistical tests, we found that SCN infection led to diverse yet non‐uniform changes in the phase and period of individual circadian clock genes (Figure S6E–M).

It should be noted that while the time‐course RNA‐seq analysis can provide transcriptome‐wide information, it has lower temporal resolution and sample size than the luciferase imaging assays. The latter, tracking the same root over time, inherently has greater power to detect significant circadian parameter changes. This methodological difference explains why rhythmic parameters from RNA‐seq for single genes may not always reach statistical significance.

To fully exploit the transcriptome‐wide advantage of the time‐course RNA‐seq analysis and reveal SCN's global impact on soybean rhythm, based on the robustness of the oscillatory expression pattern in the control samples (see the Methods section for more technical details) [19], we identified 391 time‐indicating genes for the construction of the molecular timetable of soybean hairy roots (Figure S6N; Supporting Information S2: Dataset 1). We compared periods, phases, and amplitudes of expression of these time‐indicating genes between the control and SCN‐treated samples at each sampling time point. Phases and amplitudes of these time‐indicating genes were pervasively altered upon SCN inoculation, while periods were only mildly affected by SCN (Figure 3D; Figure S6O). We also estimated their corresponding oscillation robustness indicators, ‐log_10_ (*p*), in the same way as we did for Figure S4B. For almost all the sampling time points, SCN infection perturbed the global rhythms by reducing the robustness of oscillation (Figure 3E). Taken together, the most noticeable impact of SCN on the global rhythm of soybeans is the reduction of its robustness.

### 2.4 SCN Effector Hg4E02 Is a Transcriptional Regulator Targeting Multiple Soybean Circadian Clock Gene Promoters

In the arms race between plants and pathogens, diverse kinds of pathogens have capitalized on the stockpiling of effector proteins, a specialized and fast‐evolving weapon, to interfere with the plant immune system and promote virulence [20]. Considering the importance and universality of the circadian control of the plant immune system, SCN may also use effectors to affect the soybean circadian clock. Since the expression of *GmCCA1* is significantly perturbed by SCN (Figure 3B) and CCA1 is well‐known to mediate defense against various types of pathogens in *Arabidopsis* [6], we used the promoter of *GmCCA1* as a bait in a yeast one‐hybrid (Y1H) screen to search for SCN effectors that can regulate the expression of *GmCCA1* transcriptionally.

**FIGURE 4:**
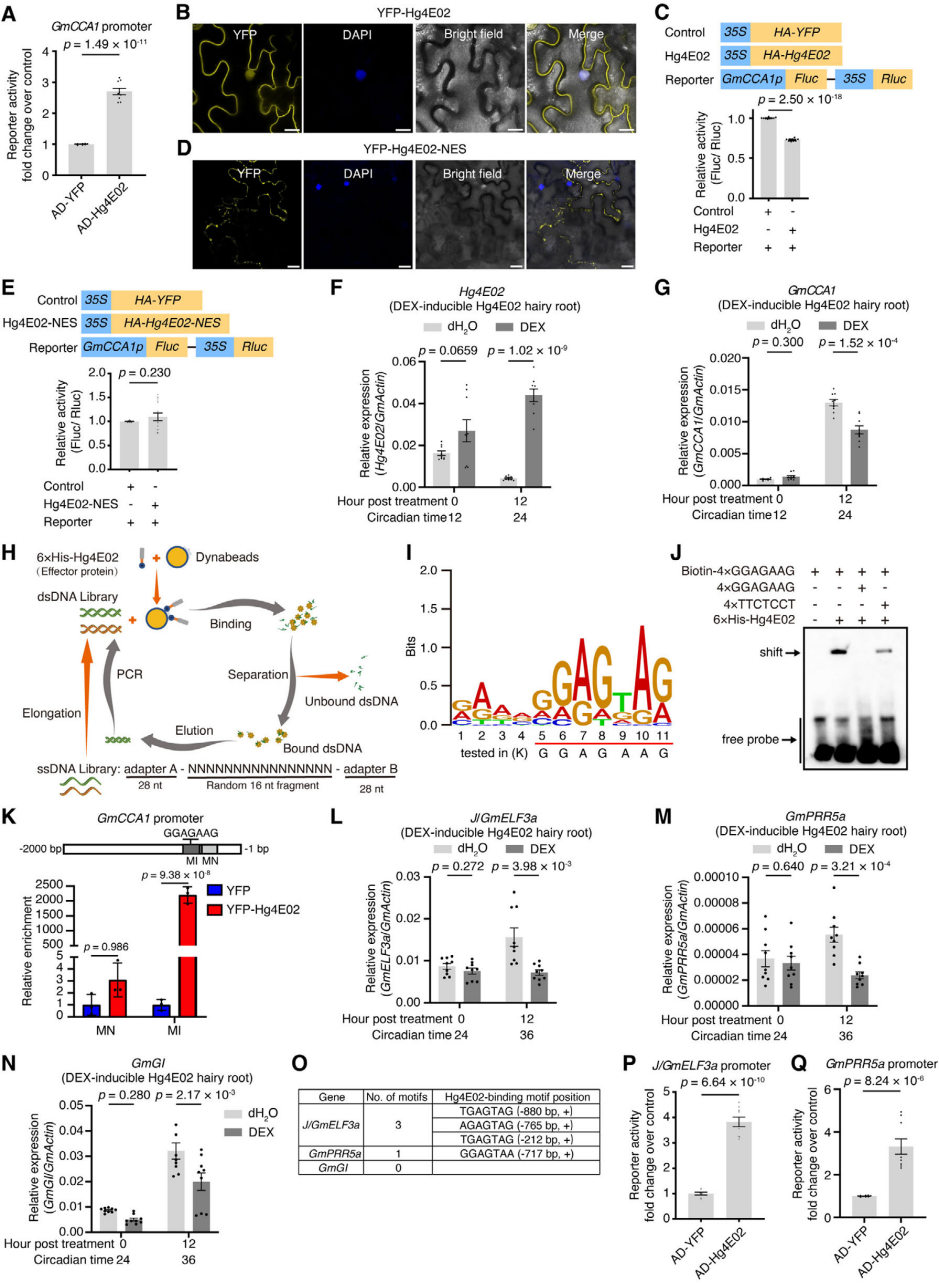
Hg4E02 directly binds to promoters of soybean circadian clock genes to repress gene expression. (A) Hg4E02 binds to the promoter of *GmCCA1* in yeast one‐hybrid assays. OD_420 nm_ was measured, and β‐galactosidase reporter activities are shown as fold change of Hg4E02 (AD‐Hg4E02) over the control (AD‐YFP) in yeast strains with the *GmCCA1* promoter. The data are shown as mean ± SEM (*n* = 3 biological replicates with 3 technical replicates per sample). The *p* value was calculated using two‐sided Student's *t‐*test. (B) Confocal microscopy images of *N. benthamiana* leaves transiently expressing YFP‐Hg4E02. Hg4E02 without signal peptide was fused to YFP. DAPI staining was performed to indicate the nucleus. Scale bars, 20 µm. This experiment was repeated three times with similar results. (C) Dual‐luciferase assay performed using *N. benthamiana* leaves transiently co‐expressing HA‐Hg4E02 or HA‐YFP (Control) and the reporter driven by *GmCCA1* promoter. The ratio of firefly luciferase (Fluc) and *Renilla* luciferase (Rluc) activities was calculated and normalized to the control. The data are shown as mean ± SEM (*n* = 4 biological replicates with 3 technical replicates per sample). The *p‐*value was calculated using two‐sided Student's *t*‐test. (D) Confocal microscopy images of *N. benthamiana* leaves transiently expressing YFP‐Hg4E02‐NES (nuclear export signal). Hg4E02 nuclear localization sequence KKPK was replaced by NES. Hg4E02‐NES without signal peptide was fused to YFP. DAPI staining was performed to indicate the nucleus. Scale bars, 20 µm. This experiment was repeated three times with similar results. (E) Dual‐luciferase assays performed using *N. benthamiana* leaves transiently co‐expressing HA‐Hg4E02‐NES or HA‐YFP (Control) and the reporter driven by *GmCCA1* promoter. The ratio of firefly luciferase (Fluc) and *Renilla* luciferase (Rluc) activities was calculated and normalized to the control. The data are shown as mean ± SEM (n = 5 biological replicates with 3 technical replicates per sample). The *p‐*value was calculated using two‐sided Student's *t*‐test. (F,G) Quantitative real‐time PCR analysis of *Hg4E02* (F) and *GmCCA1* (G) transcript abundance in transgenic soybean hairy roots expressing dexamethasone (DEX)‐inducible *Hg4E02*, treated with dH_2_O or DEX (50 µM) at the subjective dusk (CT12). Samples were harvested at 0 (CT12) and 12 (CT24) hours post treatment. *GmActin* was used as an internal control. The data are shown as mean ± SEM (*n* = 3 biological replicates with 3 technical replicates per sample). The *p* values were calculated by Holm‐Šídák's multiple comparisons test. (H) Illustration of SELEX experiment for identification of Hg4E02‐binding motifs. Refer to the Materials and Methods section for more technical details. nt, nucleotide. (I) The consensus motif identified using MEME analysis of the SELEX‐seq data. Sequences at positions 5–11 were selected for further confirmation. (J) Validation of the binding between 6×His‐Hg4E02 protein and biotin‐labeled DNA probes through EMSA. 200‐fold unlabeled DNA was used as the competitor, and the mutated competitor was used as a control. This experiment was repeated three times with similar results. (K) Validation of the binding between YFP‐tagged Hg4E02 protein and the promoter regions of *GmCCA1* through DAP‐qPCR. The data are shown as mean ± SEM from three independent experiments. The *p* values were calculated by Holm‐Šídák's multiple comparisons test. MI, the promoter region with the Hg4E02‐binding motif. MN, the promoter region without the Hg4E02‐binding motif. (L–N) Quantitative real‐time PCR analysis of *J/GmELF3a* (L), *GmPRR5a* (M), and *GmGI* (N) transcript abundance in transgenic soybean hairy roots expressing DEX‐inducible *Hg4E02* treated with dH_2_O or DEX at the subjective dawn (CT24). Samples were harvested at 0 (CT24) and 12 (CT36) hours post‐treatment. *GmActin* was used as an internal control. The data are shown as mean ± SEM (*n* = 3 biological replicates with 3 technical replicates per sample). The *p* values were calculated by Holm‐Šídák's multiple comparisons test. (O) Hg4E02 binding motifs discovered in the promoters of *J/GmELF3a*, *GmPRR5a*, and *GmGI*. (P,Q) Hg4E02 binds to the promoter of *J/GmELF3a* (P) and *GmPRR5a* (Q) in yeast one‐hybrid assays. OD_420 nm_ was measured, and β‐galactosidase reporter activities are shown as fold change of Hg4E02 (AD‐Hg4E02) over the control (AD‐YFP) in yeast strains with the *GmCCA1* promoter. The data are shown as mean ± SEM (*n* = 3 biological replicates with 3 technical replicates per sample). The *p* values were calculated using two‐sided Student's *t‐*test.

Based on previous studies that predicted potential SCN effectors [21, 22, 23], we cloned 46 SCN candidate effectors for Y1H screen (Figure S7A) and found that Hg4E02 (Hetgly.G000007551) could bind to the promoter of *GmCCA1* in yeast (Figure 4A). Bioinformatics analysis of the protein sequence of Hg4E02 revealed an exocrine signal peptide (SP) at the N‐terminus and a nuclear localization signal (NLS) at the C‐terminus (Figure S7B). The location of these motifs correlated with the three‐dimensional structure of Hg4E02 predicted using AlphaFold2 (Figure S7C). To test whether the SP of Hg4E02 (SP^Hg4E02^) can mediate the secretion of Hg4E02, which is a key feature of functional effectors, we utilized a widely used yeast secretion system [24]. In‐frame fusion of *SP^Hg4E02^* was able to mediate the secretion of SUC2, a truncated invertase without its own SP (Figure S7D,E). SP^Hg4E02^‐mediated secretion of SUC2 converted raffinose to simple sugars, which allowed the corresponding yeast strain (SP^Hg4E02^) to grow on the YPRAA medium (Figure S7D). Secreted SUC2 also generated insoluble red colored 1,3,5‐triphenylformazan (TPF), which enabled quantification of the activities of SP^Hg4E02^ (Figure S7E). The SP of *Phytophthora sojae* effector Avr1b was used as a positive control. These results suggested that SP^Hg4E02^ is functional and Hg4E02 is a secreted protein.

To further explore the function of Hg4E02 in plant cells, we constructed the truncated Hg4E02 without SP^Hg4E02^, which was referred to as Hg4E02 in short, in the following experiments. Hg4E02 fused to YFP (YFP‐Hg4E02) localized to the cytoplasm and nucleus when transiently expressed in tobacco (*Nicotiana benthamiana*) leaves (Figure 4B) or soybean hairy roots (Figure S7F). Since Hg4E02 could bind to the promoter of *GmCCA1* in yeast, Hg4E02 may regulate plant gene expression mainly through nuclear Hg4E02. Through a dual‐luciferase assay, we found that Hg4E02 repressed transcription of *GmCCA1* (Figure 4C). To further confirm the regulatory role of nuclear Hg4E02, we replaced NLS of Hg4E02 (KKPK) with the nuclear export signal (NES, LALKLAGLDI) [25] to restrict the localization of Hg4E02 within cytoplasm (Figure 4D). This Hg4E02‐NES could not regulate the transcription of *GmCCA1* (Figure 4E), supporting that nuclear Hg4E02 has a regulatory effect on gene expression.

To investigate the effects of Hg4E02 on *GmCCA1* transcription in the soybean, we generated transgenic soybean hairy roots expressing dexamethasone (DEX)‐inducible Hg4E02. The mock (dH_2_O) or DEX treatment was applied at the subjective dusk after a one‐day transition under the constant condition. The expression of *Hg4E02* could be robustly induced in soybean hairy roots 12 h after DEX treatment (Figure 4F). The expression of *GmCCA1* was suppressed by Hg4E02 at 12 h post‐treatment (Figure 4G). To interrogate the function of Hg4E02 during the natural infection of SCN on soybean, we generated transgenic soybean hairy roots carrying an RNA interference (RNAi) cassette targeting *Hg4E02*. In these root cells, small interfering RNAs (siRNAs) targeting *Hg4E02* could trigger host‐induced gene silencing (HIGS) of *Hg4E02* in SCN (Figure S7G). Using transgenic soybean hairy roots expressing the HIGS cassette targeting Hg4E02 (HIGS‐Hg4E02) or the empty vector as a negative control (Control), we analyzed the expression of *GmCCA1* in these hairy roots with mock or SCN treatment. Our results showed that SCN‐triggered suppression of *GmCCA1* in control hairy roots was abolished in HIGS‐Hg4E02 hairy roots (Figure S7H), suggesting that Hg4E02 contributes to SCN‐triggered suppression of *GmCCA1* under natural condition.

To identify the DNA elements recognized by Hg4E02, we performed systematic evolution of ligands by exponential enrichment coupled with next‐generation sequencing (SELEX‐seq) (Figure 4H). Purified 6×His‐Hg4E02 was incubated with a double‐stranded DNA (dsDNA) library containing 16 mers of random nucleotides flanked by adapters. The binders were amplified and used as the library for the next round of SELEX. After 5 rounds of selections, the dsDNA library was sequenced, and consensus motifs were identified using MEME [26] (Figure 4I). To validate the binding between Hg4E02 and the motifs identified through SELEX‐seq, we performed electrophoretic mobility shift assays (EMSA). Hg4E02 could bind GGAGAAG (Figure 4J), GGAGTAG (Figure S7I), and GGAGTAA (Figure S7J), which could be blocked by unlabeled probes but not by mutated ones. Therefore, we named these three motifs (GGAGAAG, GGAGTAG, and GGAGTAA) as Hg4E02‐binding motifs. Importantly, GGAGAAG was identified in the promoter of *GmCCA1*. To further confirm the binding between Hg4E02 and the promoter of *GmCCA1*, we performed DNA affinity purification (DAP)‐qPCR [27], which uses purified protein and plant genomic DNA to determine the potential binding region of the protein within the plant genome. The *GmCCA1* promoter region encompassing the validated Hg4E02‐binding motif (GGAGAAG) could be significantly enriched by Hg4E02, while the nearby region lacking the Hg4E02‐binding motif could not (Figure 4K). Similar enrichment of the *GmCCA1* promoter with the Hg4E02‐binding motif was also confirmed in the transgenic soybean hairy roots by ChIP‐qPCR analysis (Figure S7K). Moreover, Hg4E02's repressive effect on *GmCCA1* depends on the presence of the Hg4E02‐binding motif as revealed by dual‐luciferase analysis using the mutated *GmCCA1* promoter with the Hg4E02‐binding motifs disrupted (Figure S7L). Taken together, Hg4E02 is an SCN effector with transcriptional regulatory activity that represses transcription of *GmCCA1* through directly binding to its promoter.

Besides *GmCCA1*, we wondered whether Hg4E02 also repressed the expression of other soybean clock genes. Induction of Hg4E02 by DEX in soybean hairy root significantly suppressed expression of *J/GmELF3a*, *GmPRR5a*, and *Glycine max GIGANTEA* (*GmGI*, GLYMA_20G170000) (Figure 4L–N). Furthermore, Hg4E02‐binding motifs were identified in the promoters of *J/GmELF3a* and *GmPRR5a* (Figure 4O), indicating Hg4E02 may directly bind to the promoters of *J/GmELF3a* and *GmPRR5a*. Y1H results showed that Hg4E02 could bind to the promoters of *J/GmELF3a* and *GmPRR5a* (Figure 4P,Q). Since the promoter of *GmGI* does not contain Hg4E02‐binding motif (Figure 4O), the observed Hg4E02‐triggered *GmGI* suppression (Figure 4N) may be an indirect effect of Hg4E02, probably by affecting the expression of regulators of *GmGI*. Collectively, our results suggested that Hg4E02 suppresses the expression of multiple soybean clock genes through both direct and indirect mechanisms.

Hg4E02's transcriptional repression effects on several soybean clock gene transcription may result in changes in soybean clock rhythms. Using the molecular timetable method [10], we found that Hg4E02 induced by DEX in hairy roots did not significantly affect the period and phase of the global circadian rhythm (Figure S8). This result indicates that although Hg4E02 dampens the expression of several soybean clock genes, it has marginal effects on the global circadian rhythm. Therefore, the SCN‐triggered perturbation of clock rhythms (Figure 3E) is caused by a complicated interaction between SCN and soybean, probably involving multiple SCN effectors, immune hormones, and/or pathogen‐associated molecular patterns (PAMPs). Studies of the *Arabidopsis* circadian clock revealed that overexpression of a single clock gene usually shows a stronger circadian clock‐related phenotype than the mutant of the same clock gene [28]. This phenomenon is well supported by our findings that GmCCA1ox perturbed the soybean clock (Figures S3 and S4) while lower expression of GmCCA1 triggered by Hg4E02 did not dramatically affect the global rhythm (Figure S8).

### 2.5 Hg4E02 Represses Defense Gene Expression to Enhance SCN Virulence

**FIGURE 5:**
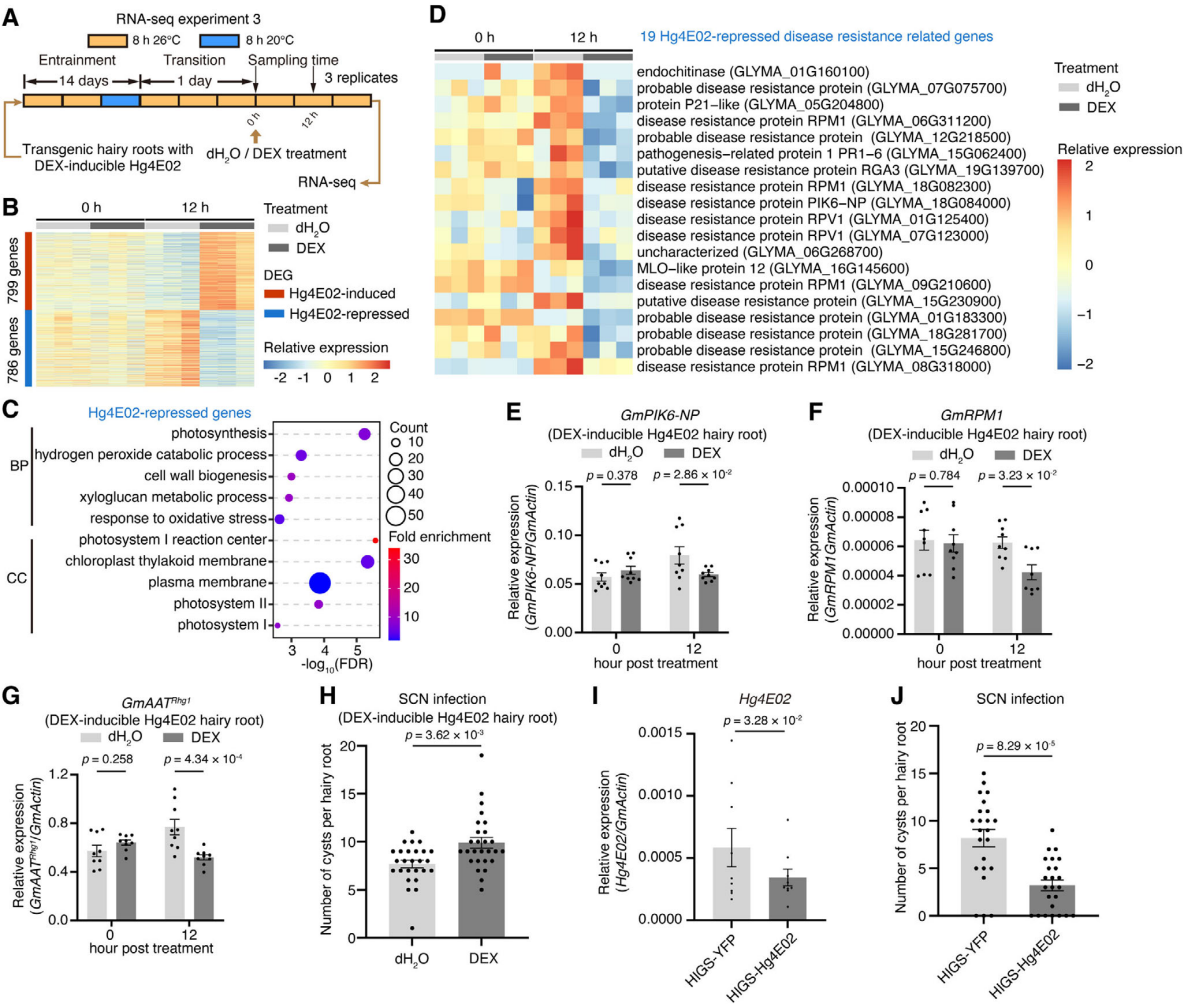
Hg4E02 represses soybean defense gene expression to enhance SCN virulence. (A) Illustration of the RNA‐seq experimental design of dexamethasone (DEX)‐inducible *Hg4E02* transgenic soybean hairy roots. Three biological replicates were obtained for the RNA‐seq analysis. (B) Expression heatmap of differentially expressed genes (DEG) induced or repressed by Hg4E02 (12 h DEX vs 12 h dH_2_O, empirical Bayes test, adjusted *p* value <0.05). (C) Enriched GO terms of biological processes (BP) and cellular components (CC) of the 786 Hg4E02‐repressed genes. FDR, false discovery rate. (D) Expression heatmap of Hg4E02‐repressed disease resistance‐related genes. (E–G) Quantitative real‐time PCR analysis of *GmPIK6‐NP* (E), *GmRPM1* (F), and *GmAAT^Rhg1^* (G) transcript abundance in transgenic soybean hairy roots expressing DEX‐inducible *Hg4E02* treated with dH_2_O or DEX (50 µM). Samples were harvested at 0 and 12 h post‐treatment. *GmActin* was used as an internal control. The data are shown as mean ± SEM (*n* = 3 biological replicates with 3 technical replicates per sample). The *p* values were calculated using Holm‐Šídák's multiple comparisons test. (H) The number of cysts on each transgenic soybean hairy root expressing DEX‐inducible *Hg4E02* treated with dH_2_O or DEX (50 µM). The data are shown as mean ± SEM (*n* = 26–27 biological replicates). The *p‐value* was calculated using the Mann‐Whitney test. (I) Quantitative real‐time PCR analysis of *Hg4E02* transcript abundance in SCN‐infected soybean hairy roots transformed with the host‐induced gene silencing (HIGS) cassette targeting *YFP* (HIGS‐YFP) or *Hg4E02* (HIGS‐Hg4E02). The data are shown as mean ± SEM (*n* = 3 biological replicates with 3 technical replicates per sample). The *p‐value* was calculated using two‐sided Student's *t‐*test. (J) The number of cysts on each transgenic soybean hairy root expressing HIGS‐YFP or HIGS‐Hg4E02. The data are shown as mean ± SEM (*n* = 26–27 biological replicates). The *p‐*value was calculated using the Mann‐Whitney test.

Since Hg4E02 affects the expression of several clock genes, it may also affect other gene expression and trigger soybean transcriptome reprogramming. Our circadian time‐course RNA‐seq of SCN infection (Figure 3C) could capture the expression pattern of SCN genes, including *Hg4E02*. The significant and sustained induction of *Hg4E02* was detected as early as 8 h after inoculation (Figure S9A). Such an early and persistent induction implied that Hg4E02 may play a role in assuring the virulence of SCN. To investigate the effects of Hg4E02 on the soybean transcriptome, we performed RNA‐seq experiments using soybean hairy roots expressing DEX‐inducible *Hg4E02*. The mock (dH_2_O) or DEX treatment was applied at the subjective dawn after a one‐day transition under the constant condition. Samples were harvested immediately or 12 h after treatment for RNA‐seq analysis (Figure 5A). The expression of *Hg4E02* could be robustly induced in soybean hairy roots 12 h after DEX treatment (Figure S9B). The multidimensional scaling (MDS) plot of the RNA‐seq data suggested good reproducibility of the 3 biological replicates (Figure S9C). Differential expression analysis identified 799 Hg4E02‐induced genes and 786 Hg4E02‐repressed genes (Figure 5B, Figure S9D,E and Supporting Information S2: Dataset 1).

GO enrichment analysis of Hg4E02‐repressed genes suggested that Hg4E02 represses defense‐related pathways such as cell wall biogenesis and response to oxidative stress (Figure 5C, Supporting Information S2: Dataset 1). Importantly, Hg4E02 also has a profound repressive impact on defense‐related genes including many *R* genes (Figure 5D). Furthermore, we performed RT‐qPCR to analyze the expression of two *R* genes, *GmPIK6‐NP* and *GmRPM1* and confirmed the repressive effect of Hg4E02 on the expression of these genes (Figure 5E,F). Hg4E02 also repressed the expression of the well‐known SCN‐resistance gene, *GmAAT^Rhg1^* (Figure 5G). These gene expression results suggest that Hg4E02 is a virulent effector that represses soybean defense gene expression to enhance SCN virulence. DEX‐inducible Hg4E02 soybean hairy roots displayed enhanced disease susceptibility to SCN upon DEX treatment (Figure 5H). To further confirm the role of Hg4E02 on SCN virulence, we used transgenic soybean hairy roots expressing HIGS‐Hg4E02 or HIGS‐YFP (Figure 5I). Soybean hairy roots expressing HIGS‐Hg4E02 showed enhanced disease resistance against SCN compared to the control soybean hairy roots expressing HIGS‐YFP (Figure 5J). Collectively, our results suggested that Hg4E02 represses soybean defense gene expression to enhance SCN virulence.

### 2.6 Antagonism Between GmCCA1 and Hg4E02 on Soybean Global Translation

**FIGURE 6:**
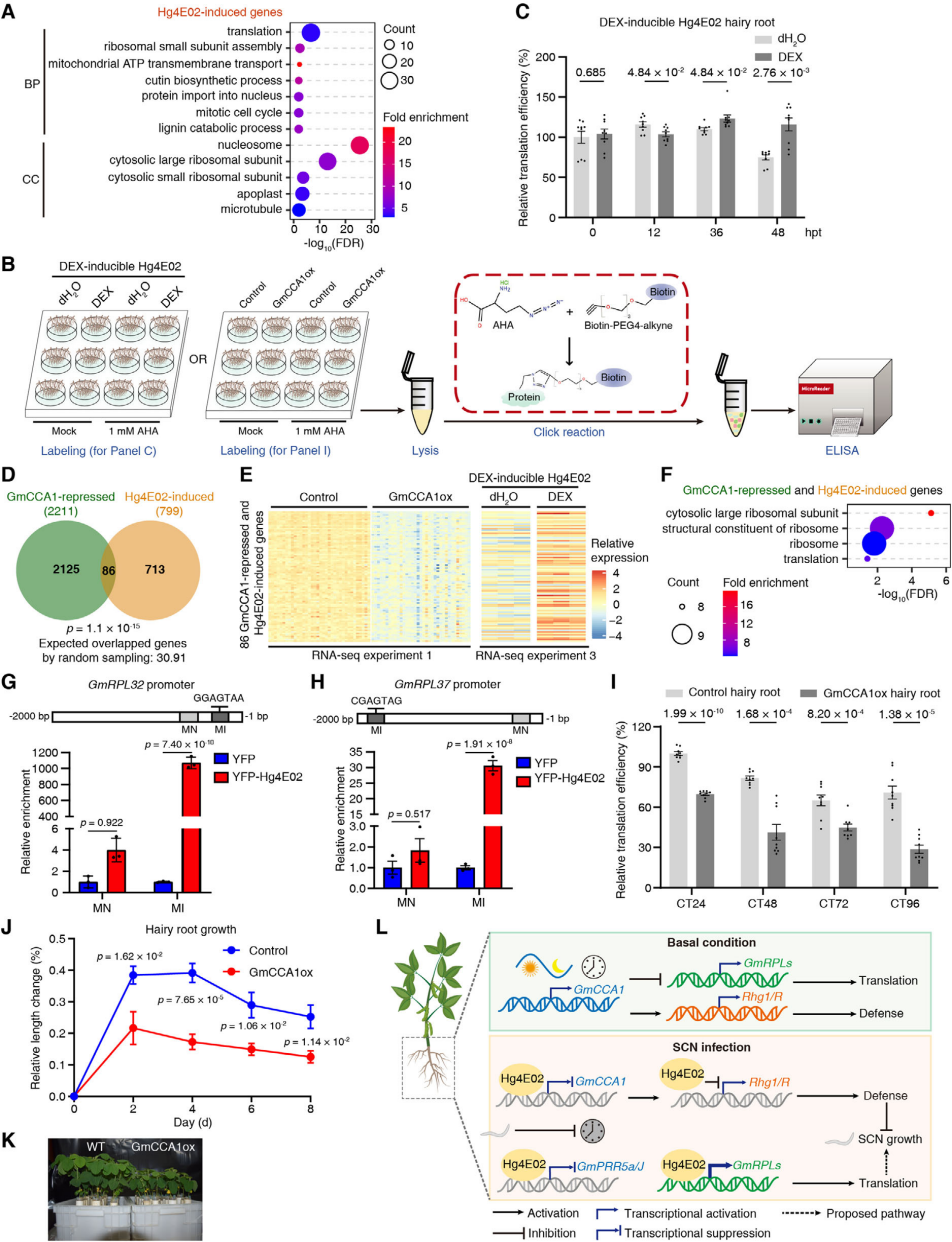
Antagonism between GmCCA1 and Hg4E02 on soybean global translation. (A) Enriched GO terms of biological processes (BP) and cellular components (CC) among 799 Hg4E02‐induced genes. FDR, false discovery rate. (B) Illustration of BONCATE in transgenic soybean hairy roots. Refer to the Methods section for more details. Labeling (For Panel C) is related to (C). Labeling (for Panel I) is related to (I). (C) Quantification of relative translation efficiency of DEX‐inducible Hg4E02 hairy roots. Hairy roots were treated with 50 µM DEX (treatment) or dH_2_O (control) for the indicated durations, and then labeled with 1 mM AHA. Data represent mean ± SEM (*n* = 9, from 3 biological replicates with 3 technical replicates). The *p* values were calculated using two‐way ANOVA followed by Holm‐Šídák's multiple comparisons test. hpt, hour post treatment. (D) Venn diagram showing the significant overlap between GmCCA1‐repressed genes and Hg4E02‐induced genes. The *p‐*value was calculated using Fisher's exact test. The expected number of overlapped genes by random sampling is shown to further demonstrate the significant overlap. (E) Expression heatmap of the overlapped 86 genes repressed by GmCCA1 and induced by Hg4E02. (F) Enriched GO terms among the overlapped 86 genes repressed by GmCCA1 and induced by Hg4E02. FDR, false discovery rate. (G,H) Validation of the binding between Hg4E02 and the promoter regions of *GmRPL32* (G) and *GmRPL37* (H) that were repressed by GmCCA1 and induced by Hg4E02 through DAP‐qPCR. The data are shown as mean ± SEM from three independent experiments. The *p* values were calculated using two‐way ANOVA followed by Holm‐Šídák's multiple comparisons test. MI, the promoter region with the Hg4E02‐binding motif. MN, the promoter region without the Hg4E02‐binding motif. (I) Quantification of relative translation efficiency of GmCCA1ox hairy roots. Hairy roots were labeled with 1 mM AHA at the time points as indicated. Data represent the mean ± SEM (*n* = 9, from 3 biological replicates with 3 technical replicates). The *p* values were calculated using two‐way ANOVA followed by Holm‐Šídák's multiple comparisons test. CT, circadian time. (J) Relative growth rate of Control and GmCCA1ox soybean hairy roots. The data are shown as mean ± SEM (*n* = 8 biological replicates). The *p* values were calculated by Holm‐Šídák's multiple comparisons test. (K) Representative photograph of 14‐day‐old WT and *GmCCA1* overexpression (GmCCA1ox) transgenic soybean seedlings. (L) Working model illustrating the interplay between SCN and soybean root circadian clock.

In addition to Hg4E02‐repressed genes based on RNA‐seq experiment 3 (Figure 5A), we also performed GO enrichment analysis of Hg4E02‐induced genes and identified significant enrichment of translation‐related and nucleosome‐related genes (Figure 6A; Supplementary Dataset 1). Soybean genes encoding histone H3 and H4, large ribosomal subunit, small ribosomal subunit, as well as other translation‐related components, were significantly induced by Hg4E02 (Figure S10). Since the syncytium, the specialized feeding site of SCN, is known to be metabolically hyperactive [16], Hg4E02‐mediated induction of translation‐related genes may contribute to the expression and metabolic reprogramming of soybean cells to fulfill the specific nutritional needs of SCN.

To obtain a dynamic view of Hg4E02's effect on global translation, we utilized a previously developed high‐throughput method to quantify newly synthesized proteins in plants [29]. This bioorthogonal non‐canonical amino acid tagging (BONCAT) and enzyme‐linked immunosorbent assay (ELISA) method (BONCATE) combines the less toxic azidohomoalanine (AHA) labeling strategy and the high‐throughput ELISA detection approach. As a structural analog of methionine, AHA can be incorporated into the newly synthesized proteins without inhibition of the peptide chain elongation. The azido group on AHA allows subsequent covalent labeling of newly synthesized proteins with the biotin–alkyne through the Click reaction and high‐throughput quantification through ELISA (Figure 6B). Consistent with the gene expression results (Figure S10), induction of Hg4E02 by DEX in soybean hairy roots started to upregulate global translation 36 h post‐treatment (hpt) (Figure 6C).

We noticed that GO analysis of GmCCA1‐repressed genes based on RNA‐seq experiment 1 (Figure 2E) identified significant enrichment of genes encoding large ribosomal subunits (Figure S5D; Supporting Information S2: Dataset 1). These results implied functional antagonism between GmCCA1 and Hg4E02. To test this hypothesis, we performed Fisher's exact tests to check whether GmCCA1‐repressed genes significantly overlap with Hg4E02‐induced genes (Figure 6D). Similarly, we also tested whether GmCCA1‐induced genes significantly overlap with Hg4E02‐repressed genes (Figure S11A). Statistically significant overlaps could be observed in these two comparisons, suggesting the partial antagonism between GmCCA1 and Hg4E02.

To reveal the soybean physiological processes that are contested by GmCCA1 and Hg4E02, we performed GO enrichment analysis using the overlapped gene lists derived from the above comparisons. GO analysis of 110 GmCCA1‐induced Hg4E02‐repressed genes (Figure S11B) found enrichment of genes involved in phenylpropanoid biosynthesis and cell wall biogenesis/degradation, which are related to plant defense (Figure S11C). GO analysis of 86 GmCCA1‐repressed and Hg4E02‐induced genes (Figure 6E; Supporting Information S2: Dataset 1) found enrichment of genes encoding ribosomal proteins (Figure 6F). To test whether Hg4E02 could bind to the *RPLs* gene promoters, we performed DAP‐qPCR using the promoters of *GmRPL32* and *GmRPL37* that were transcriptionally repressed by GmCCA1 (Figure S5G,H) and induced by Hg4E02 (Figure S10). Both genes contain Hg4E02‐binding motifs in their promoters. Hg4E02 could bind to the promoter regions containing the Hg4E02‐binding motifs (Figure 6G,H). Moreover, dual‐luciferase assays suggested that GmCCA1 repressed while Hg4E02 induced the expression of *GmRPL32* and *GmRPL37* (Figure S12). Consistent with GmCCA1's repression on the expression of translation‐related genes, soybean hairy roots overexpressing *GmCCA1* indeed suppressed global translation revealed by BONCATE assays (Figure 6B,I). Furthermore, *GmCCA1* overexpression hairy roots displayed slower root growth (Figure 6J). *GmCCA1* overexpression soybean seedlings appeared shorter than the control plants (Figure 6K). Previous studies revealed that repressed translation could trigger *Arabidopsis* root growth inhibition [29]. Therefore, the growth inhibition phenotypes observed in soybean hairy roots and seedlings overexpressing *GmCCA1* (Figure 6J,K) may also be associated with the repressed translation.

Almost all plant‐parasitic nematodes are obligate, feeding only on the living plant cells. This tight relationship may make the interplay between plant‐parasitic nematodes and the circadian clock of their host plants pervasive and conserved. Through phylogenetic analysis, we identified homologs of Hg4E02 in diverse types of plant‐parasitic nematodes, including BCN, potato cyst nematode (*Globodera pallida*), burrowing nematode (*Radopholus similis*), rice root‐knot nematode (*Meloidogyne graminicola*), northern root‐knot nematode (*Meloidogyne hapla*), and root lesion nematode (*Pratylenchus penetrans*) (Figure S13), indicating that various plant‐parasitic nematodes may employ Hg4E02‐like effectors to regulate host clock genes and repress host defense genes.

## 3 Discussion

*To win a war quickly takes long preparation*. This is especially true for sessile plants facing challenges from various pathogens. Plant resistance to various pathogens was demonstrated to display circadian and/or diurnal rhythms [7]. Studies from the last decade have established the plant circadian clock as an integral component of the plant immune system [6]. To interfere with the plant immune system, pathogens have evolved virulence effectors with diverse biochemical activities [20]. From the pathogen's perspective, the ultimate proof of the importance of circadian regulation of plant immunity is the identification of a conserved virulence effector that targets plant circadian clock components to mitigate clock‐mediated defense.

In this study, we identified Hg4E02 as such an evolutionarily conserved effector encoded by diverse kinds of plant‐parasitic nematodes, which targets soybean *CCA1* and enhances SCN virulence (Figure 6L). Our results unveiled the multi‐faceted roles of GmCCA1. GmCCA1 plays an important role in regulating the clock rhythms, as overexpressing *GmCCA1* reduces the robustness of the global rhythm (Figure S4). In addition, GmCCA1 maintains the resistance rhythm against SCN and enhances basal immunity levels by inducing *Rhg1*‐dependent and *Rhg1*‐independent defense gene expression, endowing transgenic soybean seedlings with enhanced SCN resistance (Figure 2). Moreover, GmCCA1 also represses global translation of soybean (Figure 6I). We found that Hg4E02 could directly bind to gene promoters, including clock genes and translation‐related *RPL* genes (Figures 4K, 6G,H). We also revealed the Hg4E02‐binding motifs (Figure 4I). Since Hg4E02 is a small protein (7.5 kDa) and could activate or inhibit gene expression, Hg4E02 probably functions with a transcriptional activator or repressor to regulate gene expression. Hg4E02 may also promote translation, suppress immunity, and interfere with the circadian clock through mechanisms other than transcriptional regulation. A previous study revealed that 4E02 from BCN could suppress *Arabidopsis* defense by interacting with a defense protease to alter its subcellular localization [30], suggesting that Hg4E02 may also interact with defense proteins to repress soybean immunity. In addition to Hg4E02, GLAND4, an effector of SCN and BCN, was shown to bind DNA [31], and Hs32E03, a BCN effector, was found to regulate the expression of plant rRNA through inhibition of histone deacetylases [32]. Therefore, the evolution of effectors with transcriptional regulator activity may be an effective virulent strategy adopted by parasitic nematodes.

As a sedentary obligatory root pathogen of soybeans, SCN solely depends on the host cells for its nutritional needs. It is conceivable that SCN may modulate or hijack host translation machinery to prioritize the production of specific proteinaceous nutrition for its nourishment. While transcriptional induction of translation‐related genes by SCN was documented more than 15 years ago [33], how SCN induces these genes has not been elucidated. The discovery of Hg4E02‐mediated transcriptional regulation of translation‐related genes offers one possible mechanism (Figure 6G,H, Figure S12B,D). Furthermore, BONCATE assays were first applied in soybean plants to demonstrate that Hg4E02 indeed promotes soybean global translation (Figure 6C).

The declining effectiveness of *Rhg1* calls for an alternative source of resistance to SCN. Hg4E02 realizes repression of defense responses and induction of translation‐related genes partially through recognizing the *GmCCA1* promoter to suppress *GmCCA1* and affect its downstream gene expression. By driving *GmCCA1* expression with the *35S* promoter to escape from Hg4E02's recognition, we resurrected both *Rhg1*‐dependent and *Rhg1*‐independent defense responses, providing proof‐of‐principle of how modulation of the circadian clock components could enhance disease resistance in crops. Besides *GmCCA1*, the *PR* genes, *R* genes, and MACPF domain‐containing *HR* genes induced by GmCCA1 are also potential candidates for improving SCN resistance (Figure 2F). However, constitutive overexpression of either *GmCCA1* or defense‐related genes could result in fitness cost, sacrificing the growth of the transgenic soybean seedlings, as was observed for *GmCCA1* overexpression seedlings (Figure 6K). Previous studies in rice have demonstrated that a well‐controlled inducible expression of *NPR1* and *SNC1* through integrated use of the immune‐inducible promoter and pathogen‐responsive uORFs enhances disease resistance without compromise in fitness both in the lab and the field [34]. Similar strategies combining syncytium‐specific and SCN‐inducible promoters and uORFs might be applied to control the expression of *GmCCA1* to achieve enhanced SCN resistance without sacrificing the basal growth of soybean.

## 4 Methods

### 4.1 Nematode and Plant Growth Conditions

The SCN population used in this study was race 3 (Hg type 0). The soybean cultivar Williams 82 [35], which is susceptible to SCN race 3 [36], was used. Soybean seedlings were cultured under the diurnal condition (16 h light/8 h dark), and light intensity was set to 150 µmol·m^−2^·s^−1^. Soybean hairy roots were cultured under diurnal condition (16 h 26°C/8 h 20°C) or circadian conditions (24 h 26°C with constant dark).

### 4.2 SCN Inoculation

The second‐stage juveniles (J2s) were hatched from SCN eggs at room temperature 4‐6 days before inoculation. J2s were collected each day and stored at 4°C. For soybean hairy root inoculation, hatched J2s were concentrated in a 10 mL autoclaved glass tube at 900 *g* for 3 min. After discarding the solution, 2 mL of 0.5% (w/v) streptomycin‐penicillin and 0.1% (w/v) ampicillin‐gentamycin solution was added to the glass tube and incubated for 20 min. J2s were concentrated again and then incubated in 0.5% (w/v) chlorhexidine for 3 min, followed by at least four washes with sterilized water. Hairy roots were inoculated with approximately 200 surface‐sterilized J2s in a 50 µL volume per root using a glass dropper. For soybean seedling inoculation, approximately 400‐500 hatched J2s in 1 mL volume were inoculated per seedling using a glass dropper.

### 4.3 Vector Construction for Plant Transformation

To generate the *GmCCA1* (GLYMA_03G261800) promoter‐driven luciferase vector pTF101.1‐GmCCA1p:LUC, 2,000 bp upstream to the transcription start site of *GmCCA1* was amplified as *GmCCA1p* from genomic DNA of soybean root, and then ligated together with LUC and TEM into linear pTF101.1 (*Hin*d III and *Bam*H I digestion) using Gibson Assembly Master Mix (NEB, E2611S) according to manufacturer's protocols. To generate the *GmCCA1* overexpression (GmCCA1ox) vector pTF101.1‐GmCCA1, the full‐length coding sequence of *GmCCA1* was amplified by PCR from soybean Williams 82 root cDNA and then ligated into linear pTF101.1 (*Bam*H I digestion) using basic seamless cloning and assembly kit (TransGen, CU201) according to the manufacturer's protocols. To generate dexamethasone (DEX)‐inducible *Hg4E02* vector pTF101.1‐DEX‐Hg4E02, *Hg4E02* was cloned into the engineered destination vector pTF101.1‐DEX using the Gateway cloning kit (Invitrogen, 11791‐019) according to the manufacturer's protocols. The pTF101.1‐DEX vector was constructed by ligating the DEX fragment from pTA7001‐DEST into the pTF101.1 vector using a basic seamless cloning and assembly kit. To generate transgenic soybean lines, *Agrobacterium tumefaciens* EHA105 carrying pTF101.1‐GmCCA1 was transformed into Williams 82 as described previously [37]. To generate hairy roots expressing target proteins, pTF101.1‐GmCCA1p:LUC, pTF101.1‐GmCCA1, or pTF101.1‐DEX‐Hg4E02 was transformed into *Agrobacterium rhizogenes* K599 and then transformed into soybean hairy roots as described previously [38]. All primers used were included in Supplementary Dataset 2.

### 4.4 Hairy Roots Culture and Harvest

Transformed hairy roots were root‐tip propagated on MXB medium plates with 5 mg L^−1^ Basta and 238 mg L^−1^ TIMENTIN [39]. After Basta selection, hairy roots were transferred to MXB plates with 238 mg L^−1^ TIMENTIN and cultured under the diurnal condition. Hairy roots were then kept under the circadian condition to remove the effects of oscillatory environmental factors. The start time of the circadian condition is Circadian Time 0 (CT0). For control/GmCCA1ox hairy roots, after a one‐day transition under the circadian condition, sterilized water (Mock) or surface‐sterilized J2s (SCN) were inoculated on roots at CT24. Hairy root samples were collected every 4 h for 2 days, starting at CT24 (CT24 to CT68, 12 time points). For hairy roots with DEX‐inducible *Hg4E02*, after a one‐day transition under the circadian condition, sterilized water or 50 µM DEX was sprayed on plates at CT12. Hairy root samples were collected at CT12 (0 h post‐treatment) and CT24 (12 h post treatment).

### 4.5 RNA Extraction and RT‐qPCR

Total RNA was extracted using RNAprep Pure Plant Plus Kit (TIANGEN, DP441) according to the manufacturer's instructions. The first strand cDNA synthesis (RevertAid First Strand cDNA Synthesis Kit, ThermoFisher, K1622) and quantitative PCR (FastStart Universal SYBR Green Master, Roche, 4913850001) were performed according to the manufacturer's instructions. Unless otherwise specified, *GmActin* was used as an internal control for quantifying transcript abundance. All primers used were included in Supplementary Dataset 2.

### 4.6 RNA‐seq and Data Analysis

Total RNA was used for library construction and RNA sequencing (Novogene). Raw reads were cleaned by fastp (v0.21.0) [40] to remove sequencing adapters and low‐quality reads. Clean reads were aligned simultaneously to the *H. glycines* draft [41] and *Glycine max* v2.1 genome (https://plants.ensembl.org/Glycine_max/Info/Index) using TopHat (v2.1.1) [42]. Mapped reads were counted by StringTie (v2.1.5) [43] to obtain the raw count matrix of gene expression. Transcripts per million mapped reads (TPM) were calculated as normalized read count. To identify differentially expressed genes (DEGs), R packages limma [44] and edgeR [45] were used to analyze the raw count matrix according to the protocols as described [46]. For the comparison between GmCCA1ox and Control (RNA‐seq experiment 1), the large number of samples used for the statistical tests (36 samples for OE and WT, respectively) has dramatically enhanced the detection sensitivity. To focus on the top‐ranked DEGs, adjusted *p* values < 10^−13^, a very stringent criterion was selected as the cutoff, which identified 2,181 GmCCA1‐induced genes and 2,211 GmCCA1‐repressed genes. For DEGs from the comparison between WT_SCN and WT_Control (RNA‐seq experiment 2), adjusted *p* values <0.05 were used as the cutoff, which identified 2505 SCN‐induced genes and 2467 SCN‐repressed genes. For DEGs from the comparison between 12h_DEX and 12h_dH_2_O (RNA‐seq experiment 3), adjusted *p* values <0.05 were used as the cutoff, which identified 799 Hg4E02‐induced genes and 786 Hg4E02‐repressed genes. Gene ontology (GO) enrichment analysis was performed using DAVID [47]. Soybean ENSEMBL gene IDs were converted to ENTREZ gene IDs using the Gene ID Conversion tool in DAVID. Then ENTREZ gene IDs were used for enrichment analysis. FDR < 0.05 was used to obtain significantly enriched GO terms.

### 4.7 Time‐Indicating Genes Identification and Molecular Timetable Analysis

The TPM expression matrix of genes from time‐course WT control samples (RNA‐seq experiment 1) was used to identify soybean root time‐indicating genes. For each gene, fast Fourier transform (FFT)‐nonlinear least‐squares (NLLS) analysis was performed to estimate the amplitude, period, phase, and robustness of oscillation. First, each gene was detrended to remove the linear relationship between time and expression levels. Then, FFT filtering was applied to expression levels by mapping time signals from time‐space to frequency space and then cutting higher frequencies out to re‐map back into time space. This step would greatly lower noise when fitting curves. Finally, curve fitting and parameter estimation were performed as described previously [10]. Genes with a correlation coefficient >0.7 were identified as time‐indicating genes of the soybean root. Molecular timetable analysis was performed according to our previous studies [10, 48].

### 4.8 Luciferase Imaging

Transgenic soybean hairy roots were root‐tip propagated and entrained under the diurnal condition for 6 days on MXB medium plates. The plates were then transferred to the circadian condition. The start time of the circadian condition is Circadian Time 0 (CT0). One day before imaging, hairy roots were sprayed with sterilized 2.5 mM luciferin (Gold Biotechnology) in 0.02 % Triton X‐100 (Sigma) to deplete accumulated luciferase. At CT24, hairy roots were transferred into a dark room for imaging using a CCD camera, which collected bioluminescence signals at a 1‐hour interval. At CT72, hairy roots were inoculated with an equal volume of sterile water (Mock) or surface‐sterilized J2s (SCN) at 1‐2 cm above the root tip and then returned to the dark room for imaging. Data were analyzed using Fiji.

### 4.9 Protein Structure Prediction

The protein sequence of Hg4E02 was used as input to the AlphaFold2 structure prediction model [49, 50] using the non‐docker version of AlphaFold2 (v2.0.0) (https://github.com/kalininalab/alphafold_non_docker) with the default settings. The protein structure prediction process consists of five steps, including MSA construction, template search, inference with five models, model ranking based on mean predicted local distance difference test (pLDDT), and constrained relaxation of the predicted structures [49]. The results for the models with the highest pLDDT scores, indicating the measure of residue‐level confidence, were selected. The resulting structures were analyzed using Chimera (v1.15) [51].

### 4.10 Y1H

The promoters of *GmCCA1, J*/*GmELF3a*, and *GmGI* were constructed into the pLacZi expression vector, respectively. The linearized pLacZi‐GmCCA1p vector was transferred into the YM4271 (MATa) yeast strain using the lithium acetate method. Hg4E02 or YFP (used as a control) was ligated into the pGADT7 vector. The pGADT7‐Hg4E02 or pGADT7‐YFP construct was transferred into the Y187 (MATα) yeast strain. The mating of the yeast containing pLacZi‐GmCCA1p and yeast containing pGADT7‐Hg4E02 or pGADT7‐YFP would generate offspring yeast that contained both promoter and protein of interest. The β‐galactosidase activity was quantified by ONPG assay [52].

### 4.11 Dual‐Luciferase Assay

The promoters of *GmCCA1*, *GmAAT^Rhg1^*, *GmSNAP18^Rhg1^*, and *GmWI12^Rhg1^* were respectively cloned into the vector LZ004 using Gibson Assembly Master Mix (NEB, E2611S), and the CDS of *Hg4E02^ΔSP^*, *Hg4E02^ΔSP^‐NES*, and *GmCCA1* were constructed into the pEarleyGate 201 vector using the Gateway cloning kit (Invitrogen, 11791‐019). Four‐week‐old tobacco leaves were co‐infiltrated with Agrobacteria GV3101 carrying pEarleyGate 201‐gene CDS and LZ004‐gene promoter, respectively. Tobacco leaves were sampled 2 days post inoculation, and the enzyme activities of firefly luciferase and *Renilla* luciferase were detected using the Dual‐Luciferase Reporter Assay System Kit (Promega, E1910) following the manufacturer's protocol.

### 4.12 Functional Validation of the Signal Peptide Secretory Activities

The signal peptide coding sequences of Hg4E02 and Avr1b (positive control) were constructed into the pSUC2 vector, respectively, and then transformed into the yeast strain YTK12 through the lithium acetate method. After 2 days of growth on the CMD‐W medium, yeast cells carrying fused pSUC2 vectors were screened on the YPRAA medium. Quantification of the activities of sucrose invertase was performed using 0.1% TTC (2,3,5‐Triphenyltetrazolium Chloride) as substrate according to the previous report [24].

### 4.13 Confocal Imaging

Four‐week‐old tobacco leaves were infiltrated with Agrobacterium strain GV3101 carrying LZ0102‐Hg4E02^ΔSP^ (*35S:YFP‐ Hg4E02^ΔSP^*) or LZ0102‐Hg4E02^ΔSP^‐NES (*35S:YFP‐ Hg4E02^ΔSP^‐NES*). Two days after inoculation, the tobacco leaves were infiltrated with 5 µg mL^−1^ DAPI solution 1 h before imaging by a Leica SP8 confocal microscope. For the hairy roots imaging, hairy roots respectively expressing these two fused proteins were propagated on MXB plates for 7 days. Subsequently, root tips were cut and fixed in 4% paraformaldehyde (in 1× PBS) for 30 min. After fixation, root tips were stained by 5 µg mL^−1^ DAPI solution for 1 h and washed for three times with 1× PBS before imaging by Andor Dragonfly High Speed Spinning Disk Confocal Microscope.

### 4.14 SELEX‐seq and Motif Discovery

The 72‐nt single‐strand DNA library was synthesized with a 16‐nt degenerate sequence, and short sequence adapters were added to be compatible with the Illumina platform. The sequence of adapter A is 5’‐CTTTCCCTACACGACGCTCTTCCGATCT‐3’, and the sequence of adapter B is 5’‐AGATCGGAAGAGCACACGTCTGAACTCC‐3’. The initial library was used as the template to generate the dsDNA library. The dsDNA band was recovered after separation by agarose gel electrophoresis (QIAGEN QIAquick Extraction Kit, 169016033). The CDS of *Hg4E02* was cloned into pET28a (*Bam*H I digested) using basic seamless cloning and assembly kit (TransGen, CU201). Then, the recombinant plasmid was transformed into *Escherichia coli* Rossetta (DE3) pLysS competent cells, and the fusion protein 6×His‐Hg4E02 was purified through the ÄKTA Start system. In the screening process, the mixture of Dynabeads (Invitrogen Dynabeads His‐Tag Isolation & Pulldown, 00624746) and 6×His‐Hg4E02 was incubated with the dsDNA library (5 pmol) in 500 µL SELEX Buffer (10 mM Tris‐HCl, 50 mM NaCl, 1 mM MgCl_2_, 0.02% (V/V) Tween‐20, pH 7.5) with gentle rotation at room temperature. The beads were subsequently washed with SELEX Buffer three times. After the last wash, the beads‐protein‐dsDNA complex was resuspended in 100 µL TE buffer. PCR reactions were performed to amplify dsDNA as the DNA library for the next round. The screening processes were repeated another four times. Barcodes and sequencing adapters for Illumina sequencing were added after the last round of screening. All primers used are included in Supplementary Dataset 2. Raw sequences were processed to obtain random 16‐nt sequences by removing primers and barcode sequences. All the sequences were subsequently counted to generate unique sequences sorted by the sum of each sequence. Sequences with a sum > 100 were selected as input for MEME (https://meme‐suite.org/meme) to discover novel motifs. The frequency score matrix of the top‐ranked motif from MEME was used to scan for the promoter sequence of *GmCCA1* and discover potential binding motifs.

### 4.15 DAP‐qPCR

The genomic DNA (gDNA) was extracted by CTAB from 20‐day‐old Williams 82 soybean roots. The gDNA was fragmented to an approximate size of 200 bp by sonication for library preparation. NEXTFLEX Rapid DNA Seq Kit 2.0 (Bio Scientific Corp, NOVA 5188) was used to add adapter sequences to both termini of the fragment. The sequence of adapter A is 5’‐GATCTACACTCTTTCCCTACACGACGCTCTTCCGATCT‐3’. The sequence of adapter B is 5’‐GATCGGAAGAGCACACGTCTGAACTCCAGTCAC‐3’. The initial library was then amplified by *TransStart FastPfu* Fly DNA Polymerase (TransGen, AP231‐01). The coding sequence of the YFP‐3×GGGGS tandem sequence was fused in‐frame to the N‐terminus of Hg4E02, and the recombinant sequence was cloned into pET28a. The recombinant protein was expressed in *E. coli* Rossetta (DE3), and the fusion protein was purified in the ÄKTA Start system. In the screening process, the mixture of GFP‐Trap Magnetic Agarose beads (Chromoteck, gtma‐20) and 6×His‐YFP‐3×GGGGS‐Hg4E02 was incubated with 500 ng amplified DAP library in 500 µL dilution buffer (10 mM Tris‐HCl, 150 mM NaCl, 0.5 mM EDTA, pH 7.5) with gentle rotation at 4°C for 1.5 h. Then the beads were washed three times using washing buffer [10 mM Tris‐HCl, 150 mM NaCl, 0.5 mM EDTA, 0.02% (V/V) Tween‐20, pH 7.5] at 4°C and dilution buffer for each. The bound DNA was eluted using 200 mM glycine and neutralized in 1 M Tris (pH 10.4). The eluent was amplified by PCR, and the DNA fragments of 200‐400 bp in agarose gel were recovered by QIAquick Gel Extraction Kit (QIAGEN, 28706) for generation of the DNA library for the next round of selection. 6×His‐YFP alone was purified as a negative control. After 5 rounds of screening, the libraries were used as templates for qPCR, and the library that was not screened was used as an input control.

### 4.16 ChIP‐qPCR

Approximately 1 g of soybean hairy roots were cut into 2–5 mm segments, crosslinked in 1% formaldehyde for 20 min, and then subjected to vacuum treatment for 5 min in the presence of 125 mM glycine. Samples were washed 2‐3 times with ddH_2_O. The dried samples were then fast‐frozen and ground in liquid nitrogen. To obtain nuclei, samples were lysed on ice for 10 min in 25 mL of Extraction buffer 1 (10 mM Tris‐HCl pH 8.0, 1 mM EDTA, 250 mM sucrose, 10 mM KCl, 40 mM NaCl, 0.1% SDS, 1% Triton X‐100, 1 mM PMSF, and 1× Protease inhibitors). The lysate was filtered through a 40‐µm mesh into a new 50‐mL tube and centrifuged at 3000 g for 15 min at 4°C. The pellet was washed twice with Extraction buffer 2 (10 mM Tris‐HCl pH 8.0, 1 mM EDTA, 0.1% SDS, 1% Triton X‐100, 1 mM PMSF, and 1× Protease inhibitors). The obtained nuclei were then lysed in 500 µL of Nuclei Lysis Buffer (10 mM Tris‐HCl pH 8.0, 1 mM EDTA, 0.2% SDS, 1 mM PMSF, and 1× Protease inhibitors) by sonication. After centrifugation at 12 000 rpm for 10 min at 4°C, the supernatant was transferred to a new 1.5‐mL tube. 5% of the supernatant was saved as input, and the remaining part was pre‐cleared with 15 µL of Protein A/G beads (Thermo Fisher, 20421) for 30 min. After centrifugation, the supernatant was equally divided into two parts (control and experimental groups), and the volume was adjusted to 1 mL using Binding buffer (10 mM Tris‐HCl pH 8.0, 1 mM EDTA, 100 mM NaCl, 0.05% SDS, 1% Triton X‐100, 1 mM PMSF, and 1× Protease inhibitors). Subsequently, 50 µL of Protein A/G beads were added to each group. HA antibody (Abmart, M20003L) and Flag antibody (Vazyme, RA1003) were added to the control and experimental groups at a 1:100 dilution, respectively, followed by overnight incubation. Both groups were then washed sequentially with low salt buffer (10 mM Tris‐HCl pH 8.0, 1 mM EDTA, 150 mM NaCl, 0.1% SDS, 1% Triton X‐100, 1 mM PMSF, and 1× Protease inhibitors) for two times, high salt buffer (10 mM Tris‐HCl pH 8.0, 1 mM EDTA, 300 mM NaCl, 0.1% SDS, 1% Triton X‐100, 1 mM PMSF, and 1× Protease inhibitors) for two times, and 10 mM Tris‐HCl buffer for two times. Input, experimental, and control groups were resuspended in 70 µL of RC buffer (10 mM Tris‐HCl pH 8.0, 2 mM EDTA, 300 mM NaCl, 0.2% SDS) and incubated with 1 µL of RNase for 30 min at 37°C. Subsequently, 1 µL of Proteinase was added for reverse crosslinking at 55°C for 1 h and 63°C for 8 h. DNA was extracted using the phenol‐chloroform extraction method and subjected to qPCR.

### 4.17 EMSA

Synthesized 5’‐terminal biotin‐labeled forward and reverse single‐stranded oligonucleotides were annealed in the annealing buffer (10 mM Tris, 1 mM Na_2_EDTA, 50 mM NaCl) to generate double‐stranded probes. The EMSA experiments were performed with 50 ng protein and 50 fmol double‐stranded probes using LightShift Chemiluminescent EMSA Kit (Thermo Fisher Scientific) according to the manufacturer's instructions. The concentration of the competitive probes without the biotin label was 200‐fold that of the biotin‐labeled probes. Concentrations of competitive probes and mutated competitive probes were 10 µM.

### 4.18 Quantification of SCN on Soybean Hairy Roots

Control and GmCCA1ox hairy roots were entrained either under the diurnal condition for 7 days or the diurnal condition for 6 days with a one‐day transition under the circadian condition. Around 200 surface‐sterilized SCN J2s were inoculated on each root using a glass dropper every 4 h for 2 days under the circadian condition. After 28 days, SCN cysts were counted on each root using a stereomicroscope every 4 h for 2 days. At 4 days post inoculation, hairy roots inoculated at CT24 and CT40 were stained with acid fuchsin to visualize nematodes. The number of nematodes was counted using a stereomicroscope.

### 4.19 Quantification of SCN on Soybean Seedlings

WT and GmCCA1ox soybean plants were grown under the diurnal condition for 14 days and transferred to the circadian condition. After a one‐day transition, approximately 400–500 J2s were inoculated per seedling at dusk. After 30 days, seedlings were immersed in water to remove sand, and the number of cysts was counted using a stereomicroscope.

### 4.20 BONCATE Assay

After induction and selection, the positive transgenic *GmCCA1*‐overexpression (GmCCA1ox) and control hairy roots were propagated on MXB solid medium under a diurnal temperature cycle of 16 h at 26°C followed by 8 h at 20°C in the dark. On the seventh day post‐propagation, these hairy roots were entrained under constant temperature 26°C, starting at 8:00 a.m. At 8:00 a.m. of the eighth day (CT24), every five hairy roots were taken as a biological replicate and labeled with 1 mM AHA (Macklin, S920180), which was dissolved in MXB liquid medium, for 2 h at 26°C in the dark. Following the labeling, the hairy roots were sampled, flash‐frozen in liquid nitrogen, and then ground into a powder. Additional samples were collected at the following time points as indicated in the figures. A 1× PBS solution containing a protease inhibitor cocktail (EDTA‐free, 1:50 dilution) (Roche, 4693132001) was used as extraction buffer, and the slurry was subsequently sonicated in icy water for 40 min. The lysate was centrifuged at 18,000 *g* for 10 min at 4°C, and the total soluble protein in the supernatant was quantified using Bradford's assay.

For each click reaction, 30 µg of protein was mixed with 100 µM biotin‐alkyne (Sigma, 764213), 333 µM CuSO_4_ (Macklin, C836340), 0.5 mg mL^−1^ sodium ascorbate (Aladdin, S105024), 666 µM THPTA (Sigma, 762342), and 1× PBS to a final total volume of 60 µL in an Eppendorf tube. The tube was gently shaken in the dark at room temperature for 1.5 h. Post‐click reaction, 40 µL PBS, 400 µL methanol, 100 µL chloroform, and 300 µL water were added to terminate the reaction and facilitate protein extraction by centrifuging at 14 000 *g* for 2 min at 4°C. The protein was found in the middle layer after centrifugation. Then, 400 µL methanol was added, and the protein was precipitated by centrifuging at 14 000 *g* for 10 min at 4°C three times. The pellet was air‐dried at room temperature before being redissolved in 30 µL 2 M urea (dissolved in 1× PBS). Protein quantification was performed again using Bradford's assay. ELISA was conducted in a 96‐well high‐binding plate (Corning) coated with 0.5 µg protein (as antigen, diluted with 50 µL of PBS) per well, and incubated at 4°C overnight. Unbound antigen in each well was washed out with 200 µL of PBST three times for 5 min each at room temperature. The bound antigen was then blocked with 100 µL of 2% BSA (dissolved in PBST) for 1 h at 37°C. After discarding the blocking buffer, 50 µL streptavidin‐HRP (Abcam, diluted 1:60 000 in PBST) was added and incubated for 40 min at room temperature. Unbound antibodies in each well were washed out with 200 µL of PBST five times for 5 min each. Once the PBST was completely removed, 50 µL of HRP substrate (0.1% o‐phenylenediamine, 0.075% (V/V) H_2_O_2_, 0.1 M citric acid, 0.2 M Na_2_HPO_4_, pH 5.6 at room temperature) was added to each well and incubated for 8 min at room temperature in the dark. The reaction was terminated with 50 µL 2 M H_2_SO_4_, and the HRP signals were detected at a wavelength of 492 nm using a microplate reader (BioTek Cytation5).

### 4.21 Root Growth Measurement

Control and GmCCA1ox hairy roots were root‐tip propagated on MXB medium plates under the diurnal condition. After 8 days, hairy roots were inoculated with an equal volume of sterile water (Mock) or surface‐sterilized J2s (SCN) at 1‐2 cm above the root tip. The root tip position was marked at a two‐day interval. At 4 dpi, photos were taken, and the root growth length was measured by Fiji.

### 4.22 Phylogenetic Tree Construction

The protein sequence of Hg4E02 was used as the template to perform BLASTp searches on NCBI, EBI, and NEMBASE4. Hits with E values less than or equal to 1e‐12 were selected for downstream analysis. The protein sequences of Hg4E02 homologs were aligned using ClustalW in MEGAX (v10.1.8) [53]. The phylogenetic tree was constructed by MEGAX using the JTT+F+G model with 1000 bootstrap steps. Multiple sequence alignment was performed by Clustal X software.

### 4.23 Statistical analysis

All statistical analyses were performed using RStudio and GraphPad Prism. Multiple tests were corrected using the methods indicated in the figure legends.

## Author Contributions

XW, YX, YH, LC and RJ contributed to this study equally. Conceptualization: WW and MZ. Methodology: XW, YX, WW, LC, HP, YH, CC, RM, TM, and AS. Formal analysis: XW, YX, WW, YH, and RM. Investigation: XW, YX, LC, WW, HP, YH, RJ, CC, RM, TM, YT, YS, EL, LK, CG, WZ, PS, and WD. Visualization: XW, YX, YH, YS, WW, and MZ. Funding acquisition: WW, MZ, DP, HP, TB, and CC. Project administration: WW, MZ, DP, and TB. Supervision: WW, MZ, DP, AS, and TB. Writing – original draft: WW and MZ. Writing – review & editing: WW, MZ, XW, YX, LC, HP, YH, CC, TB, and RM

## Funding

State Key Laboratory for Gene Function and Modulation Research, School of Life Sciences, Peking University (W.W.), National Natural Science Foundation of China 31970641 (W.W.), Center for Life Sciences (W.W.), National Natural Science Foundation of China 32370288 (M.Z.), Beijing Nova Program of Science and Technology Z191100001119027 (M.Z.), Capital Normal University (M.Z.), Support Project of High‐level Teachers in Beijing Municipal Universities in the Period of 14th Five‐year Plan BPHR20220114 (M.Z.), National Natural Science Foundation of China 31972247 (H.P.), The Agricultural Science and Technology Innovation Project of the Chinese Academy of Agricultural Sciences (ASTIP‐02‐IPP‐15) (HP), National Natural Science Foundation of China 32072398 (D.P.), State of Iowa and Hatch Act Funding (IOW03808 & 4308) (W.W., T.B., T.M., R.M., A.S.), North Central Soybean Research Program (NCSRP) (T.B., A.S., T.M., R.M.), and  Postdoctoral Fellowship of the Center for Life Sciences (C.C.).

## Conflicts of Interest

M.Z., W.W., X.W., and Y.X. are listed as inventors on a pending patent application on the GmCCA1‐mediated enhancement of SCN resistance of soybean.

*Glycine max* gene symbols and corresponding gene IDs can be found in Supplementary Dataset 3. Genomic sequences of genes from this article can be found on the Soybase (https://www.soybase.org) or Phytozome (https://phytozome‐next.jgi.doe.gov). The data that support the findings of this study are available from the corresponding author upon reasonable request.;

## Supporting information




**Supporting File 1**: advs75547‐sup‐0001‐SuppMat.pdf.

**Supporting File 2**: advs75547‐sup‐0002‐DataSet.zip.

## Data Availability

Raw RNA sequencing data of time‐course control/SCN/GmCCA1ox samples (RNA‐seq experiment 1 and 2) have been deposited in the Sequence Read Archive (SRA) of the National Center for Biotechnology Information (NCBI) under project PRJNA813432. RNA‐seq data of dH_2_O/DEX samples (RNA‐seq experiment 3) have been deposited in the NCBI SRA database (PRJNA858541). SELEX‐seq data have been deposited in the NCBI SRA database (PRJNA860038).
