# Supplementary material for: Hijacking the Host Clock: A Nematode Effector Antagonizes Soybean Circadian Defense and Translation Control
Source: Adv Sci (Weinh). 2026 May 7:e18591. Online ahead of print. doi: 10.1002/advs.202518591 (PMC13334672; doi:10.1002/advs.202518591)
Supplement: Supplementary file 1 — Supporting File 1: advs75547‐sup‐0001‐SuppMat.pdf. [file ADVS-9999-e18591-s002.pdf]

Supporting Information

Hijacking the host clock: A nematode effector antagonizes soybean circadian defense and translation control

Xingwei Wang, Yufeng Xu, Yanfei Hu, Lijun Cao, Ru Jiang, Changtian Chen, Rick Masonbrink, Thomas Maier, Yuchen Tu, Yabo Shi, Enhui Liu, Lingan Kong, Chan Guo, Wei Zhao, Peng Shi, Wenzhen Du, Andrew Severin, Thomas Baum, Deliang Peng, Huan Peng, Mian Zhou*, and Wei Wang**

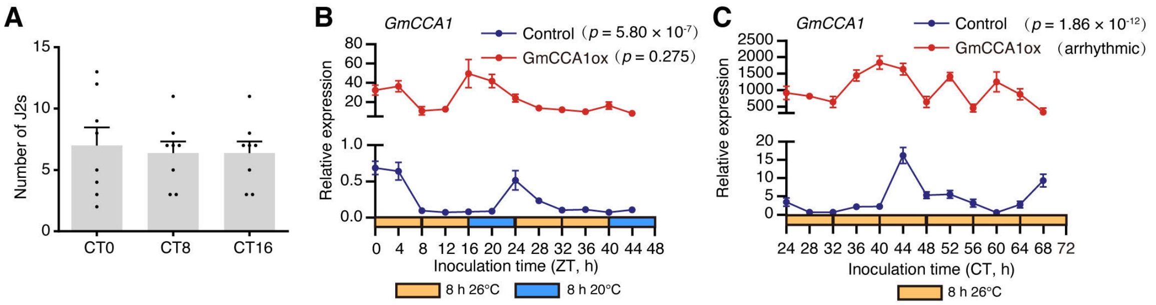

Figure S1. Validation of *GmCCA1* overexpression hairy roots. **(A)** The number of hatched J2s in circadian time (CT). All J2s were hatched from eggs under 26°C for 3 days. Data are plotted as mean ± SEM (n = 8). **(B, C)** Quantitative real-time PCR analysis of *GmCCA1* transcript abundance in Control and *GmCCA1* overexpression (GmCCA1ox) hairy roots under diurnal (**B**) and circadian (**C**) conditions. *GmActin* was used as an internal control. The data are shown as mean ± SEM (n = 3). The oscillation robustness was derived through nonlinear regression analyses and *F*-test using linear trends as null hypotheses. Control showed significant resistance rhythms under both diurnal ($p = 5.80\times10^{-7}$) and circadian ($p = 1.86\times10^{-12}$) conditions. GmCCA1ox did not show robust resistance rhythms under either the diurnal ($p = 0.275$) or circadian conditions (unable to fit a cosine wave, the *p* value is not available). ZT, zeitgeber time. CT, circadian time.

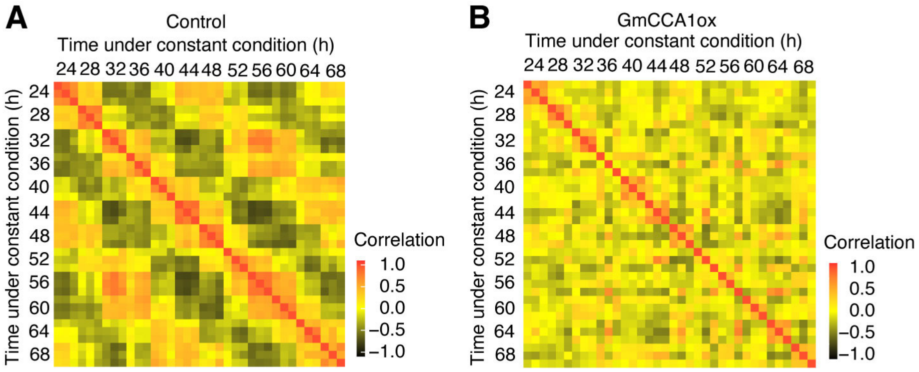

Figure S2. Quality check of the circadian time-course RNA-seq experiment. Pairwise Pearson correlation heatmaps of Control (**A**) and GmCCA1ox (**B**) samples were calculated using soybean time-indicating genes. Three biological replicates are shown as adjacent columns and rows within each sampling time point.

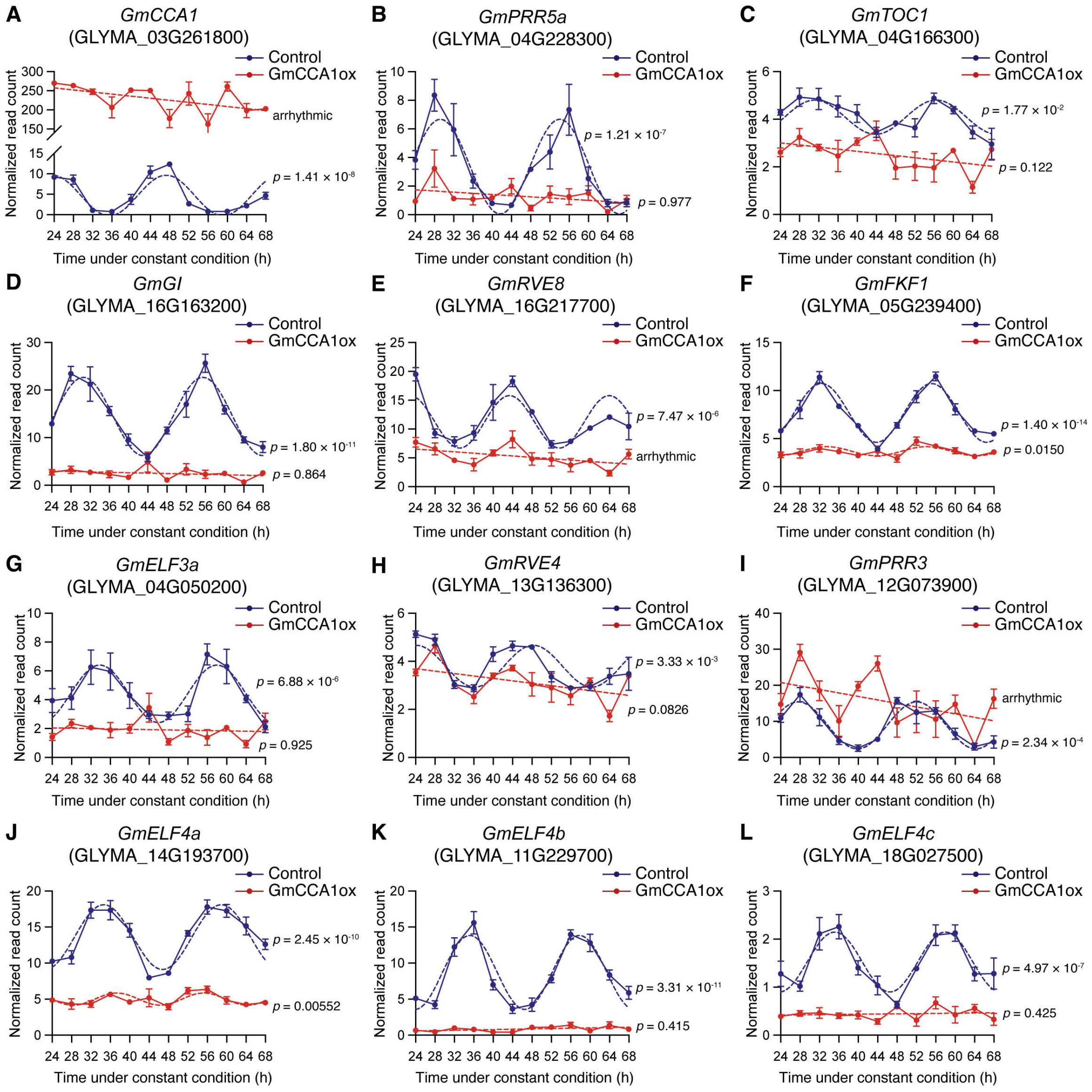

Figure S3. *GmCCA1* overexpression compromises the circadian rhythmic expression pattern of the central circadian clock genes. **(A-L)** Normalized read counts of representative central circadian clock genes in Control and GmCCA1ox soybean hairy roots. The data are shown as mean ± SEM (n = 3 biological replicates). The dashed lines represent the fitted straight lines or cosine waves from nonlinear regression analyses. The *p* values were derived from *F*-test of the model comparison between the cosine wave and the straight line. The curves that could not be fitted by a cosine wave, thus prohibiting the derivation of the *p* values through *F*-test, were labeled as “arrhythmic”.

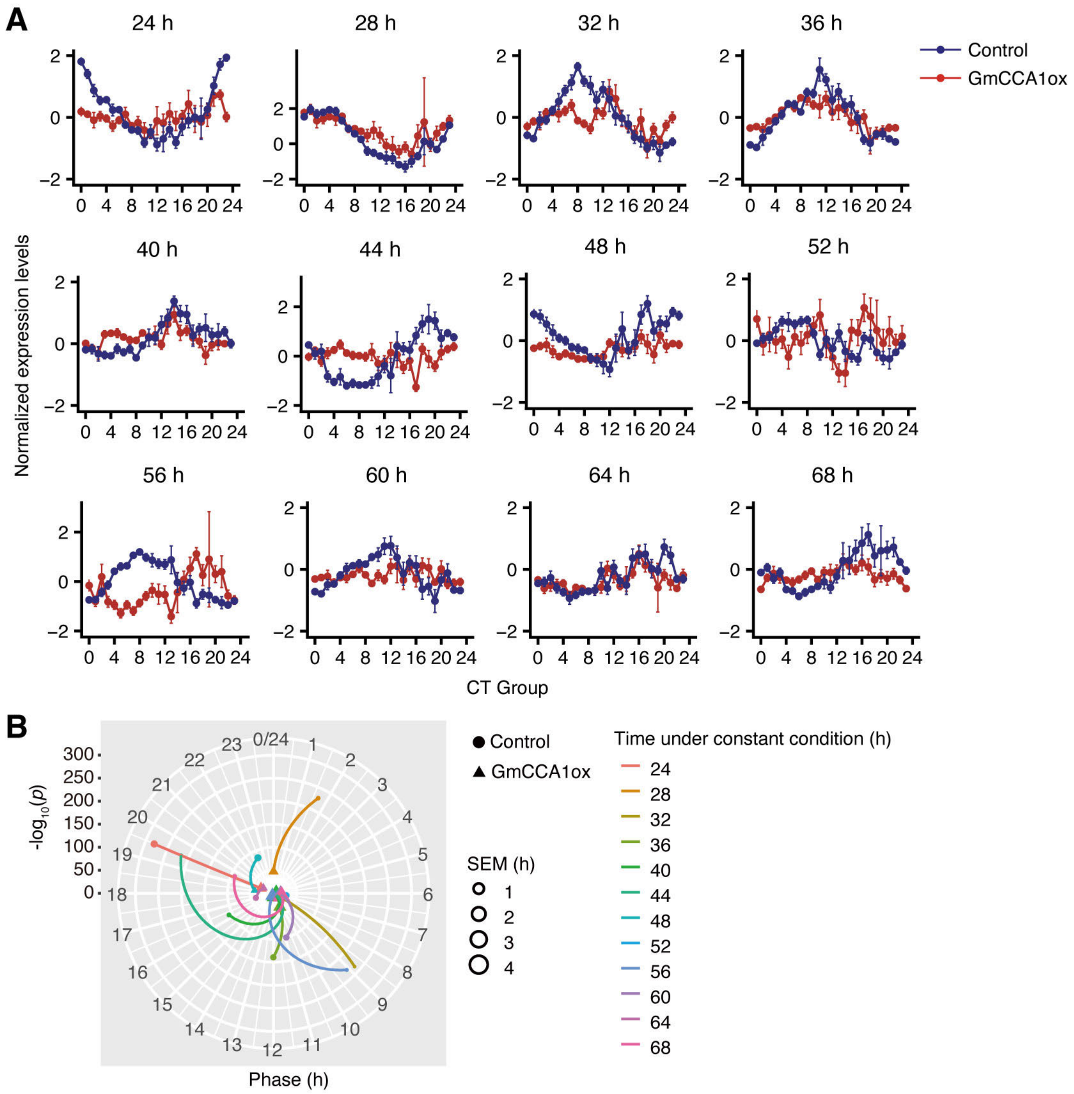

Figure S4. *GmCCA1* overexpression reduces soybean clock robustness. **(A)** Global rhythm changes caused by GmCCA1ox. Each time point is plotted as normalized expression levels of time-indicating genes binned to 24 circadian time (CT) groups according to their phase. The data are shown as mean ± SEM (n = 3 biological replicates). **(B)** Radial plot showing the phase shift and robustness changes caused by *GmCCA1* overexpression (GmCCA1ox) at each sampling time point. Phase is plotted as the angular coordinate. Robustness plotted as the radial distance is indicated by $-\log_{10}(p)$, with a larger $-\log_{10}(p)$ representing better oscillation. SEMs are indicated by the symbol size of the circles (Control) or triangles (GmCCA1ox).

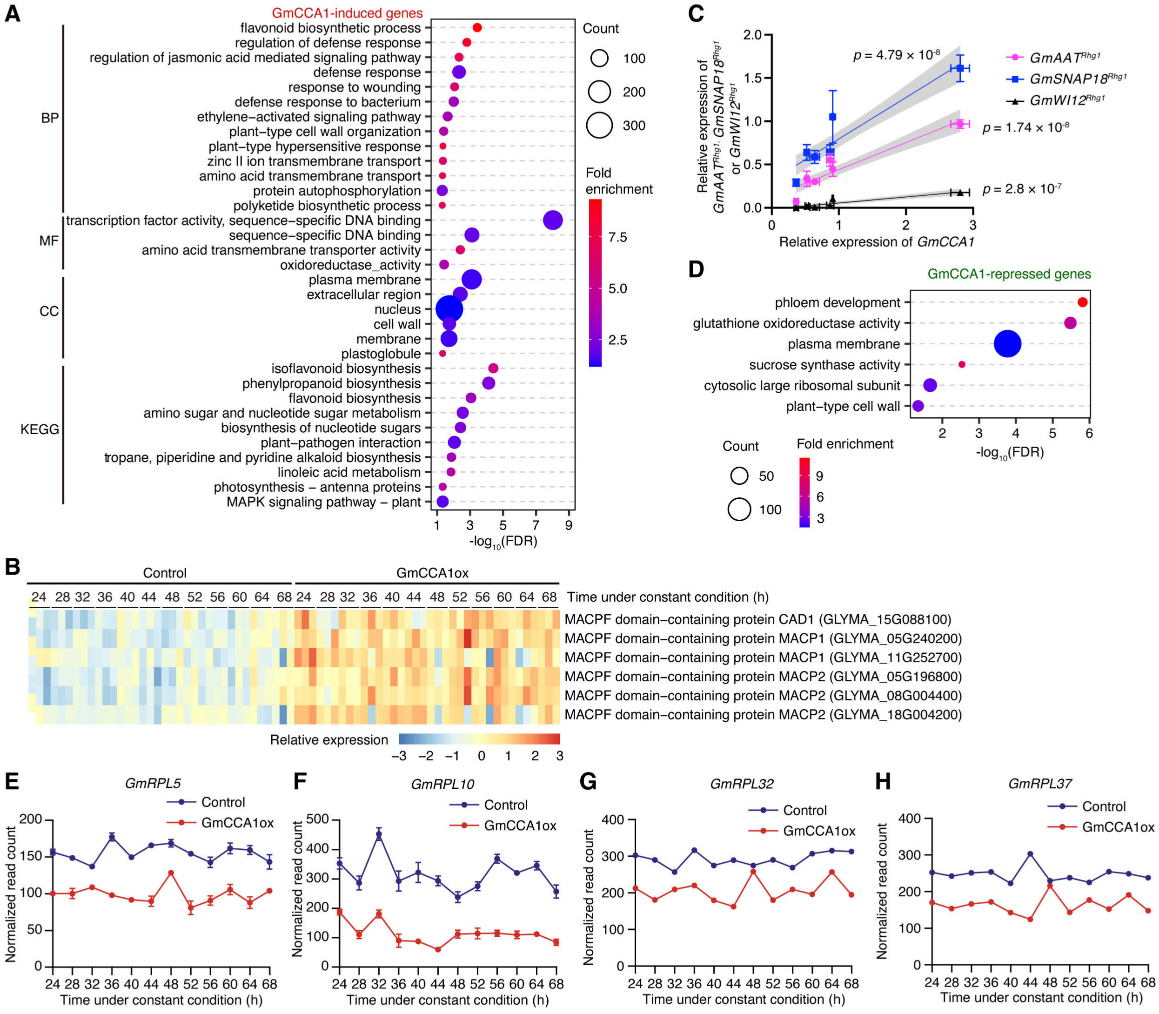

Figure S5. GmCCA1 promotes defense gene expression and suppresses translation-related gene expression. **(A)** Enriched GO terms of biological processes (BP), molecular function (MF), cellular components (CC), and KEGG pathways of GmCCA1-induced genes. FDR, false discovery rate. **(B)** Expression heatmap of GmCCA1-induced genes associated with plant-type hypersensitive responses. **(C)** Linear regression (dashed lines) of transcript abundance of *GmCCA1* with $GmAAT^{Rhg1}$, $GmSNAP18^{Rhg1}$, and $GmWI12^{Rhg1}$. Data are from quantitative real-time PCR results of WT and GmCCA1ox soybean seedlings (n = 3 biological replicates, each with 3 technical replicates). The transcript abundance of *GmCCA1* is plotted on the x-axis. The transcript abundance of $GmAAT^{Rhg1}$, $GmSNAP18^{Rhg1}$, and $GmWI12^{Rhg1}$ is plotted on the y-axis. The data are shown as mean ± SEM. The *p* values were derived from *F*-test. **(D)** Enriched GO terms of the GmCCA1-repressed genes. FDR, false discovery rate. **(E-H)** Transcript abundance of GmCCA1-repressed genes associated with translation in Control and GmCCA1ox samples. The data are shown as mean ± SEM (n = 3 biological replicates).

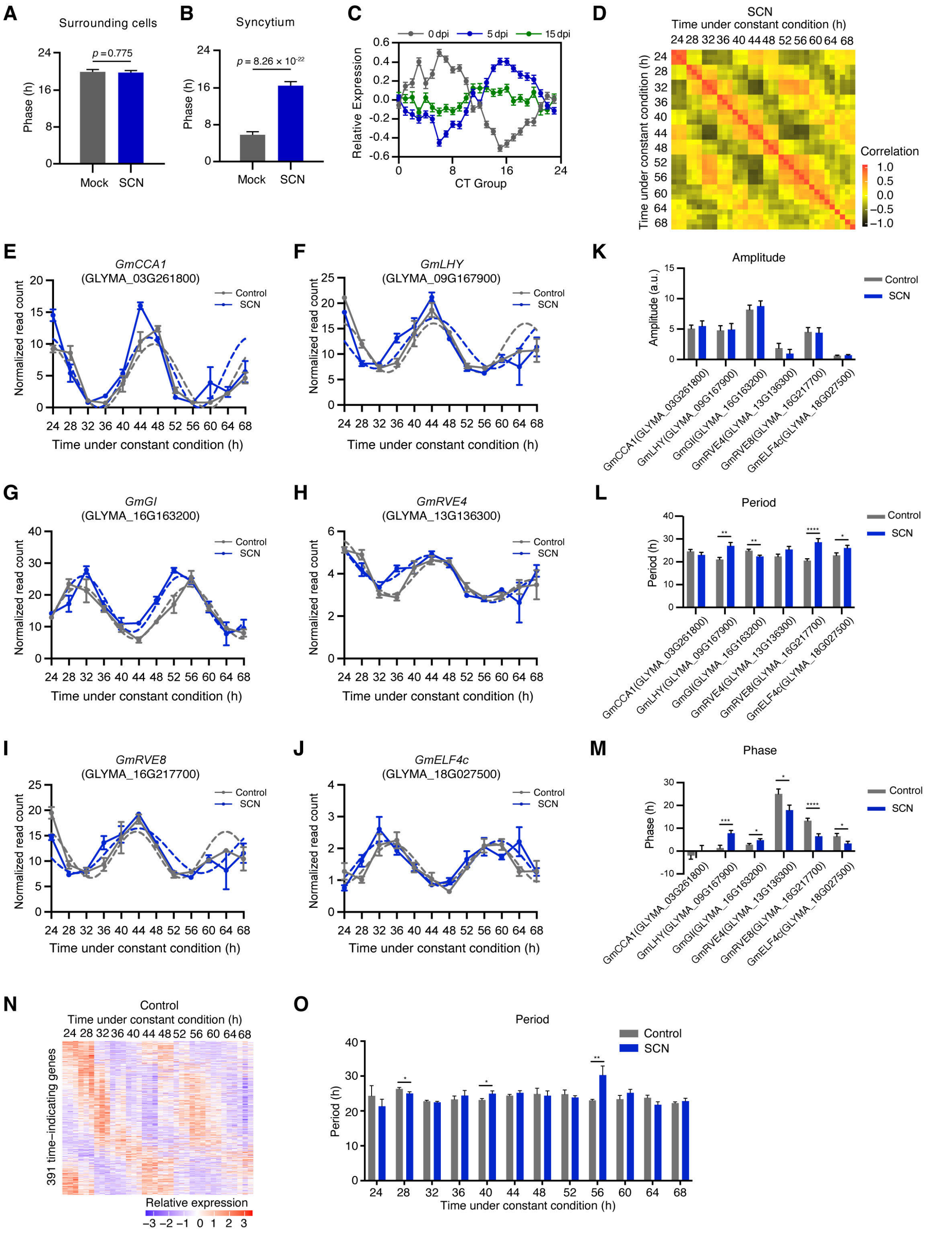

Figure S6. SCN infection affects soybean circadian clock. **(A, B)** Estimated phase of soybean global rhythm upon Mock or SCN treatment (SCN) in surrounding cells (**A**) and syncytium (**B**) according to data from Fig. 3A. The data are shown as mean ± SEM (n = 3 biological replicates). The *p* values were calculated using two-sided Student's *t*-test. **(C)** Molecular timetables of *Arabidopsis* infected by beet cyst

nematode (BCN) at 0, 5, and 15 days post inoculation (dpi). Each time point is plotted as normalized expression levels of time-indicating genes binned to 24 circadian time (CT) groups according to their phase. The data are shown as mean ± SEM (n = 3 biological replicates). **(D)** Heatmap of the pairwise Pearson correlations of the circadian time-course RNA-seq samples. Three biological replicates are shown as adjacent columns and rows within each sampling time point. (**E-J**) Normalized read counts of *GmCCA1*, *GmLHY*, *GmGI*, *GmRVE4*, *GmRVE8* and *GmELF4c* in Control and SCN-infected soybean hairy roots. The data are shown as mean ± SEM (n = 3 biological replicates each time point). The dashed lines represent the fitted cosine waves from nonlinear regression analyses. (**K-M**) Amplitude (K), period (L) and phase (M) of *GmCCA1*, *GmLHY*, *GmGI*, *GmRVE4*, *GmRVE8* and *GmELF4c* in Control and SCN infected soybean hairy roots. The data are shown as mean + SEM (n = 3 biological replicates). The amplitude, period and phase were calculated by nonlinear regression analyses. The statistical significance was determined by Holm-Šídák's multiple comparisons test (* $p < 0.05$, ** $p < 0.01$, *** $p < 0.001$, **** $p < 0.0001$). **(N)** Expression heatmap of soybean time-indicating genes. A total of 391 soybean time-indicating genes were sorted by their phase of expression from 0 to 24 h. Each row represents one time-indicating gene. Three biological replicates are shown as adjacent columns within each sampling time. **(O)** Period changes caused by SCN at 12 sampling time points. The data are shown as mean ± SEM (n = 3 biological replicates). The period of each sampling time point was calculated through the molecular timetable method. The statistical significance was determined by Holm-Šídák's multiple comparisons test (* $p < 0.05$, ** $p < 0.01$).

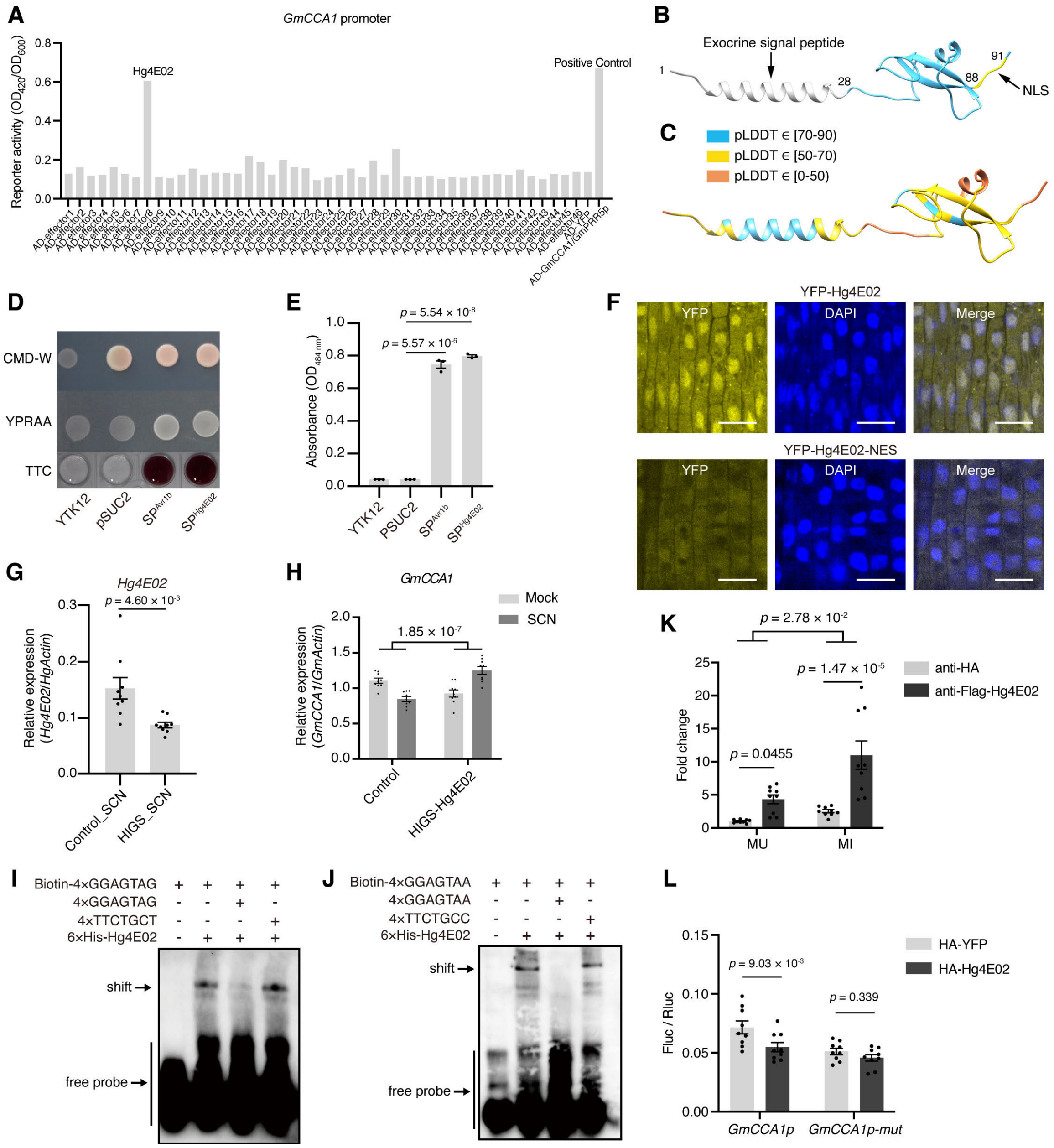

Figure S7. Hg4E02 has a functional signal peptide and DNA-binding ability. **(A)** Yeast one-hybrid screen identifying Hg4E02 as a candidate SCN effector capable of binding the promoter of *GmCCA1*. $OD_{420\ nm}$ was measured, and β-galactosidase reporter activities are shown as fold change of Hg4E02 (AD-Hg4E02) over the control (AD-YFP) in yeast strains with the *GmCCA1* promoter. **(B)** Hg4E02 protein structure predicted using AlphaFold2. Amino acid positions are indicated by numbers. The exocrine signal peptide (SP) and nuclear localization signal (NLS) are indicated by arrows. **(C)** Predicted local distance difference test (pLDDT) scores of the three-dimensional structure of Hg4E02 predicted using AlphaFold2. **(D)** Functional validation of the signal peptide of Hg4E02. Yeast YTK12 strains carrying the Hg4E02 signal peptide fragments fused in-frame to the invertase gene in the pSUC2 vector (SP^{Hg4E02}) can grow on both the CMD-W media (with sucrose,

yeast growth even in the absence of invertase secretion) and YPRAA media (with raffinose instead of sucrose, growth only when invertase is secreted), as well as reduce 2,3,5-triphenyltetrazolium chloride (TTC) to an insoluble red colored compound (1,3,5-triphenylformazan, TPF), indicating secretion of invertase. The untransformed YTK12 strain and YTK12 carrying the empty pSUC2 vector are used as negative controls. The signal peptide of effector Avr1b (SP^{Avr1b}) was used as a positive control. This experiment was repeated three times with similar results. **(E)** Quantification of TPF by recording the absorbance at 484 nm of yeast strains in the presence of 0.1% TTC. Data represent the mean ± SEM (n = 3). The *p* values were calculated using one-way ANOVA followed by Sidak's multiple comparisons test. (**F**) Confocal microscopy images of transgenic soybean hairy roots expressing YFP-Hg4E02 or YFP-Hg4E02-NES (nuclear export signal). DAPI staining was performed to indicate the nucleus. Scale bars, 20 μm. **(G)** Quantitative real-time PCR analysis of *Hg4E02* transcript abundance in SCN-infected soybean hairy roots transformed with the empty vector (Control) or the host-induced gene silencing (HIGS) cassette targeting *Hg4E02*. The transcript abundance of *HgActin* was used as an internal control. The data are shown as mean ± SEM (n = 9, from 3 independent experiments with 3 technical replicates). The *p* value was calculated using two-sided Student's *t*-test. (**H**) Quantitative real-time PCR analysis of *GmCCA1* transcript abundance in mock- or SCN-treated hairy roots transformed with empty vector (Control) or HIGS cassette targeting *Hg4E02* (HIGS-4E02) at CT36. *GmActin* was used as an internal control. The data are shown as mean ± SEM (n = 9, from 3 independent experiments with 3 technical replicates). The *p* value was calculated using two-way ANOVA. **(I, J)** EMSA validation of the binding between the His-Hg4E02 protein and two biotin-labeled DNA probes predicted using MEME. 200-fold unlabeled DNA was used as the competitor, and the mutated competitor was used as a control. These experiments were repeated three times with similar results. (**K**) ChIP-qPCR analysis showing the binding of Hg4E02 to the *GmCCA1* promoter using soybean hairy roots overexpressing Flag-Hg4E02. Anti-HA antibody was used as a negative control. Data represent the mean ± SEM (n = 3 biological replicates). The *p* values were calculated using two-way ANOVA followed by Holm-Šídák's multiple comparisons test. MI, *GmCCA1* promoter region with Hg4E02-binding motifs. MU, *GmCCA1* CDS region without Hg4E02-binding motifs. (**L**) Dual-luciferase assays performed using *N. benthamiana* leaves transiently co-expressing HA-Hg4E02 or HA-YFP and the reporter driven by *GmCCA1* promoter (*GmCCA1p*) or mutated *GmCCA1* promoter with Hg4E02-binding motifs disrupted (*GmCCA1p-mut*). The data are shown as mean ± SEM (n = 3 biological replicates). The *p* values were calculated by Holm-Šídák's multiple comparisons test.

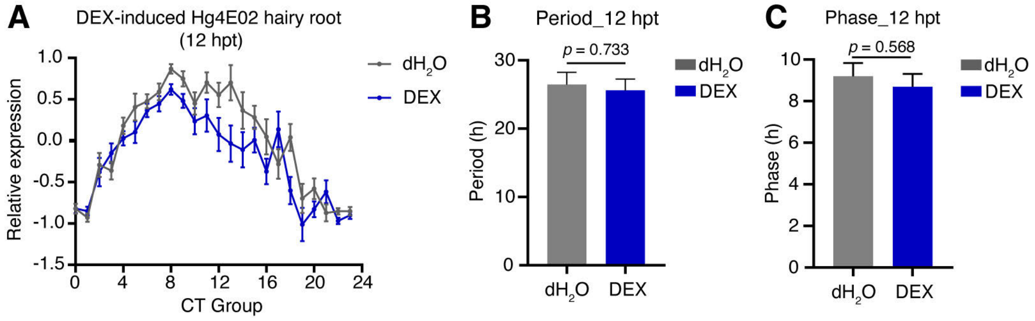

Figure S8. Hg4E02 does not affect soybean global circadian rhythm. **(A)** Normalized expression levels of time-indicating genes are plotted from DEX-induced Hg4E02 hairy roots at 12 hours post treatment (hpt) with dH_2O or DEX (50 μM). The data are shown as mean ± SEM (n = 3 biological replicates). CT, circadian time. **(B, C)** Period (**B**) and phase (**C**) derived from (**A**). The data are shown as mean ± SEM. The *p* values were calculated using unpaired *t*-test with Welch's correction.

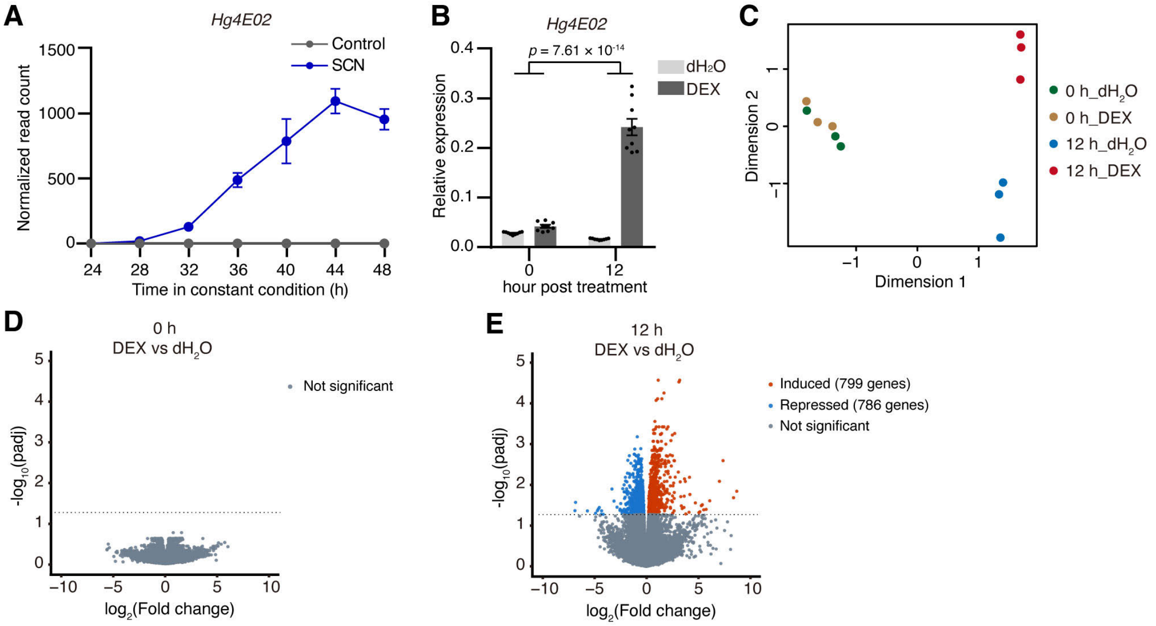

Figure S9. Analysis of RNA-seq data of DEX-inducible Hg4E02 soybean hairy roots. **(A)** Transcript abundance of *Hg4E02* in soybean hairy roots after Control or SCN treatment based on time-course RNA-seq illustrated in **Fig. 3C**. The data are shown as mean ± SEM (n = 3 biological replicates). **(B)** Quantitative real-time PCR analysis of *Hg4E02* transcript abundance in transgenic soybean hairy roots expressing DEX-inducible *Hg4E02*. dH_2O or DEX (50 μM) was treated at 0 hour. Samples were harvested at 0 and 12 hours post treatment. *GmActin* was used as an internal control. The data are shown as mean ± SEM (n = 9, from 3 independent experiments with 3 technical replicates). The *p* value was calculated using two-way ANOVA. **(C)** Multidimensional scaling plot of RNA-seq data of transgenic soybean hairy roots expressing DEX-inducible *Hg4E02* with dH_2O or DEX treatment at 0 hour. Samples were harvested at 0 and 12 hours post treatment. **(D, E)** Volcano plots showing differentially expressed genes (adjusted p value < 0.05) comparing DEX and dH_2O-treated samples at 0 hours post treatment (**D**) and 12 hours post treatment (**E**). The adjusted *p* values (padj) were calculated using empirical Bayes test.

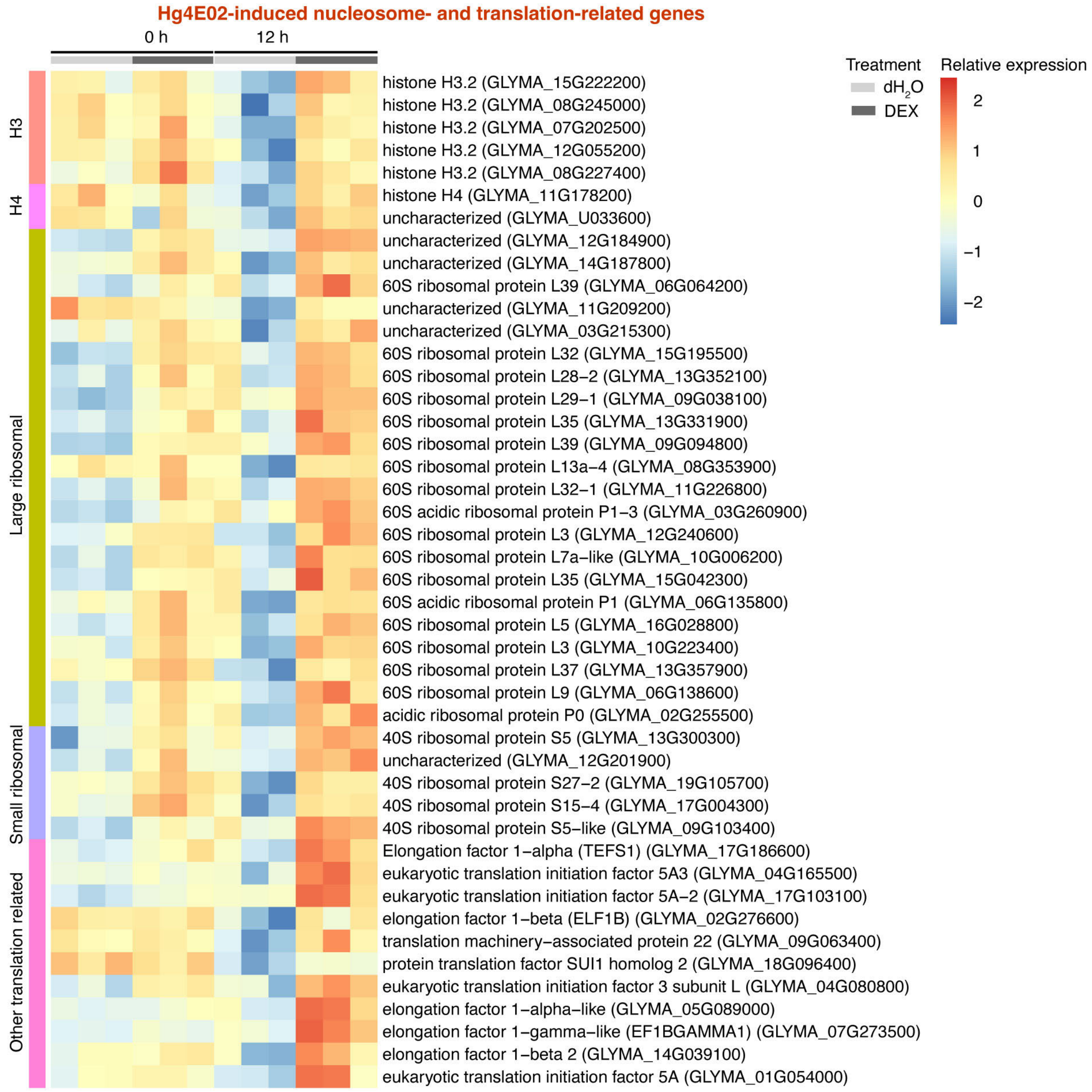

Figure S10. Hg4E02 induces translation-related gene expression. Expression heatmap of Hg4E02-induced nucleosome- and translation-related genes from RNA-seq experiment 3 illustrated in **Fig. 5A**.

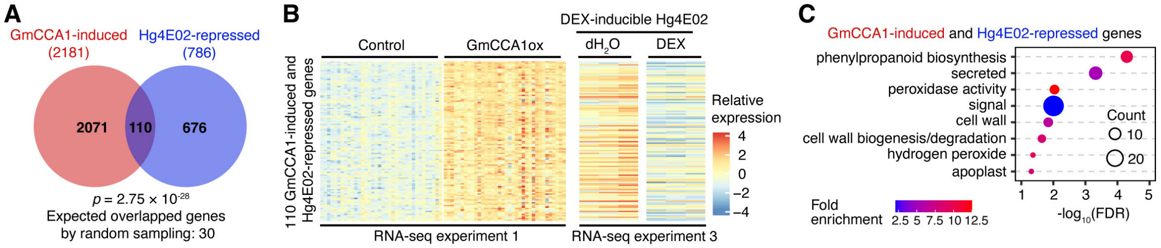

Figure S11. Analysis of GmCCA1-induced and Hg4E02-repressed genes. **(A)** Venn diagram showing the significant overlap between GmCCA1-induced genes and Hg4E02-repressed genes. The *p* value was calculated using Fisher's exact test. The expected number of overlapped genes by random sampling is shown to further demonstrate the significant overlap. **(B)** Expression heatmap of the overlapped 110 genes induced by GmCCA1 and repressed by Hg4E02. **(C)** Enriched GO terms among the overlapped 110 genes induced by GmCCA1 and repressed by Hg4E02. FDR, false discovery rate.

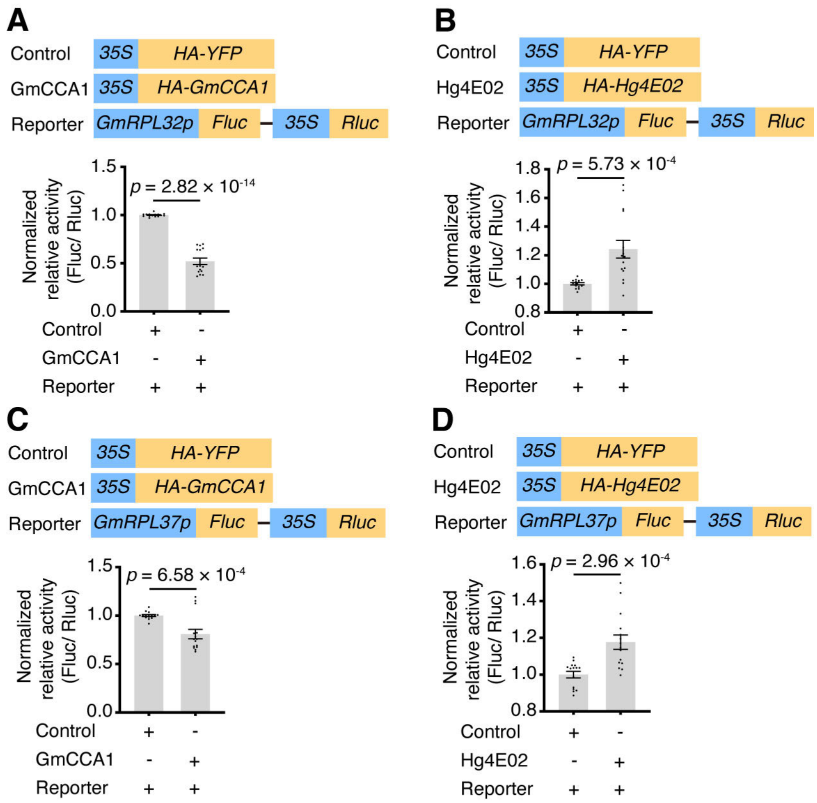

Figure S12. Transcriptional regulation of GmCCA1 and Hg4E02 on expression of GmRPLs. Dual-luciferase assays performed using *N. benthamiana* leaves transiently co-expressing HA-YFP (Control) or HA-CCA1 (**A**, **C**) or HA-Hg4E02 (**B**, **D**), and the reporter driven by *GmRPL32* promoter (**A**, **B**) or *GmRPL37* promoter (**C**, **D**). The ratio of firefly luciferase (Fluc) and *Renilla* luciferase (Rluc) activities was calculated and normalized to the control. The data are shown as mean ± SEM (n = 15, 5 independent experiments with 3 technical replicates). The *p* values were calculated using two-sided Student's *t*-test.

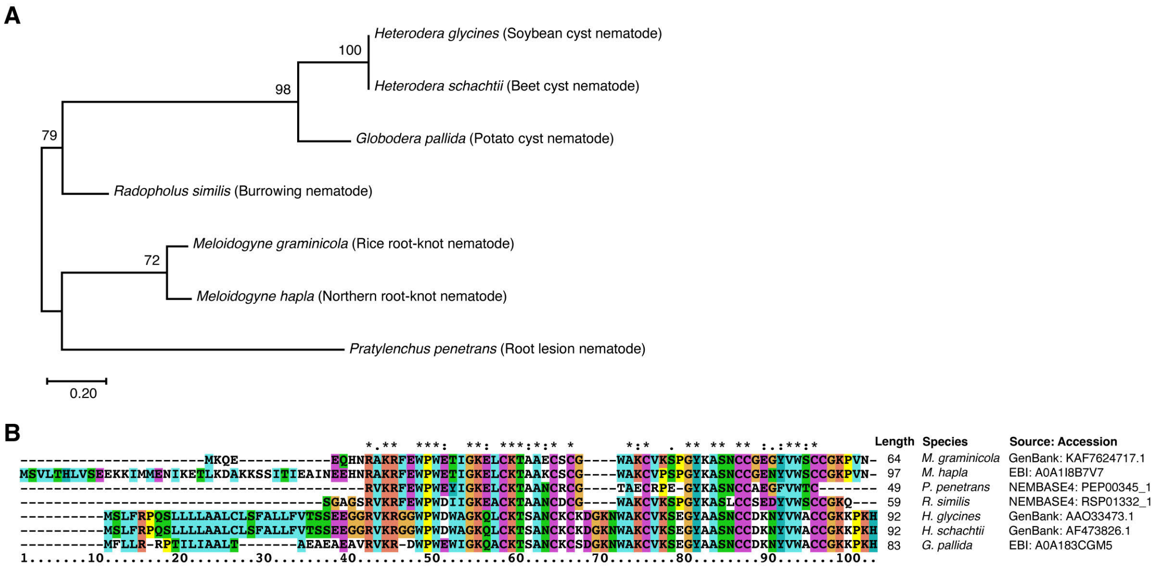

Figure S13. Phylogenetic analysis of Hg4E02 and its homologs from other plant-parasitic nematodes. **(A)** Phylogenetic analysis of the protein sequences of Hg4E02 and its homologs from other plant-parasitic nematodes. Scale bar, 0.2 amino acid substitutions per site. **(B)** Protein sequence alignment of Hg4E02 and its homologs from other plant-parasitic nematodes. Sequence sources and accessions are shown on the right panel. “*” indicates positions that have a single, fully conserved residue. “:” indicates positions that have strongly conserved residue groups. “·” indicates positions that have weak conserved residue groups.

Supplementary Dataset 1 (separate file). This file contains the information related to the RNA-sequencing data.

Supplementary Dataset 2 (separate file). This file contains the information on primers used in this study.

Supplementary Dataset 3 (separate file). This file contains information about *Glycine max* gene symbols and corresponding gene IDs used in this study.
